# Epidemiology of herpes simplex virus type 1 in the United States: Systematic review, meta-analyses, and meta-regressions

**DOI:** 10.1016/j.isci.2024.110652

**Published:** 2024-08-05

**Authors:** Rwedah A. Ageeb, Manale Harfouche, Hiam Chemaitelly, Laith J. Abu-Raddad

**Affiliations:** 1 Infectious Disease Epidemiology Group, Weill Cornell Medicine-Qatar, Cornell University, Qatar Foundation - Education City, Doha, Qatar; 2 World Health Organization Collaborating Centre for Disease Epidemiology Analytics on HIV/AIDS, Sexually Transmitted Infections, and Viral Hepatitis, Weill Cornell Medicine–Qatar, Cornell University, Qatar Foundation – Education City, Doha, Qatar; 3 Department of Population Health Sciences, Weill Cornell Medicine, Cornell University, New York, NY, USA; 4 Department of Public Health, College of Health Sciences, QU Health, Qatar University, Doha, Qatar; 5 College of Health and Life Sciences, Hamad Bin Khalifa University, Doha, Qatar

**Keywords:** Health sciences, Disease

## Abstract

This study aimed to analytically describe the epidemiology of herpes simplex virus type 1 (HSV-1) infection in the United States through a systematic review and meta-analytics. We reviewed 159 publications, identifying 190 seroprevalence measures and 43 proportions of HSV-1 detection in genital herpes. The pooled mean HSV-1 seroprevalence was 38.0% (95% CI: 30.9–45.4) among general-population children and 63.5% (95% CI: 61.3–65.7) among general-population adults. Age explained 43% of the seroprevalence variation, with rates increasing progressively with age. Seroprevalence declined by 0.99-fold (95% CI: 0.99–0.99) per year. The pooled mean proportion of HSV-1 detection in genital herpes was 15.4% (95% CI: 10.8–20.6), increasing by 1.02-fold (95% CI: 1.00–1.04) per year. Recurrent genital herpes had a 0.17-fold (95% CI: 0.09–0.32) lower proportion of HSV-1 detection compared to first-episode cases. The epidemiology of HSV-1 is shifting, marked by a decline in oral acquisition during childhood and an increase in genital acquisition during adulthood.

## Highlights

• HSV-1 seroprevalence was estimated at 38.0% among general-population children
• HSV-1 seroprevalence was estimated at 63.5% among general-population adults
• HSV-1 seroprevalence is decreasing at a rate of 1% per year
• HSV-1 as a cause of genital herpes is increasing at a rate of 2% per year

## Graphical abstract

**Figure:**
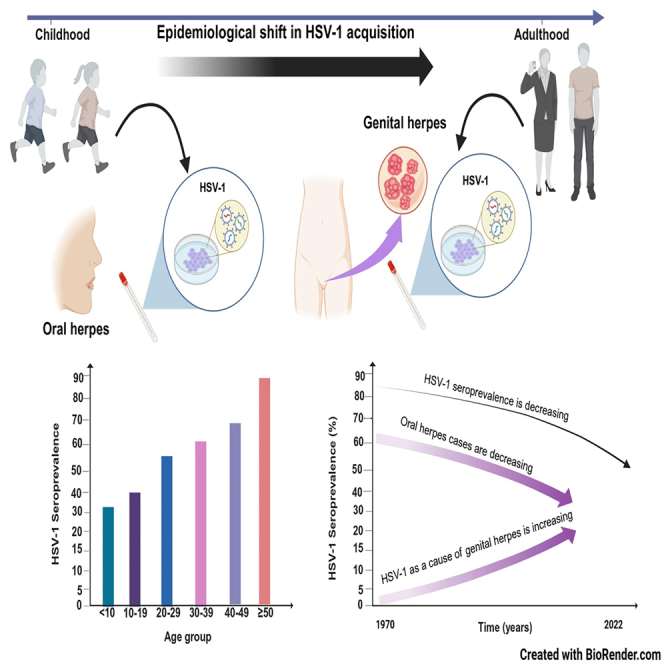

## Introduction

Herpes simplex virus type 1 (HSV-1) infection is widespread, affecting populations globally.^1^ HSV-1 establishes lifelong latency in the trigeminal ganglia,^2^ periodically reactivating and causing subclinical viral shedding.^3^^,^^4^^,^^5^^,^^6^ Typically acquired during childhood, the virus primarily spreads through direct contact with infected secretions from oral lesions or an infected person’s saliva.^7^^,^^8^ Clinical manifestations of HSV-1 infection include a diverse range of mucocutaneous, neurological, and corneal diseases, varying in severity, such as cold sores, herpetic whitlow, gingivostomatitis, meningitis, encephalitis, and corneal blindness.^7^^,^^8^ While rare, transmission from genitally infected mothers to their neonates during birth,^9^ as well as postnatal transmission through oral contact from caregivers, can lead to neonatal herpes—a disabling disease in newborns with a high fatality rate.^10^

HSV-1 can be transmitted through oral sex or sexual intercourse, both during asymptomatic or symptomatic shedding, leading to genital herpes, a subset of genital ulcer disease (GUD) that is typically caused by HSV-2 infection.^11^^,^^12^^,^^13^^,^^14^ This occurs when the HSV-1 virus gains entry through the genital area in individuals who have not been previously infected orally.^11^^,^^12^^,^^13^^,^^14^ The impact of HSV-1 and HSV-2 infections on public health and their economic costs,^1^^,^^15^^,^^16^ along with their changing epidemiology, has garnered considerable attention from the World Health Organization (WHO) and global partners who are spearheading an initiative aimed at developing preventive and therapeutic HSV vaccines.^17^^,^^18^

While systematic reviews have characterized the epidemiology of HSV-1 infection in various regions and countries,^19^^,^^20^^,^^21^^,^^22^^,^^23^^,^^24^^,^^25^ none have specifically addressed the United States of America. Accordingly, this study was conducted to characterize the epidemiology of HSV-1 infection in the United States. We investigated the seroprevalence of HSV-1 as the primary outcome of this study. Additionally, as secondary outcomes, we examined the proportion of HSV-1 detection in clinically diagnosed GUD cases and laboratory-confirmed genital herpes cases. Through an analytical meta-analysis and meta-regression approach, we examined the levels of infection and assessed the influence of specific factors on HSV-1 epidemiology, explored temporal trends, and identified potential sources of heterogeneity across studies.

## Results

### Search results and scope of evidence

**Figure 1:**
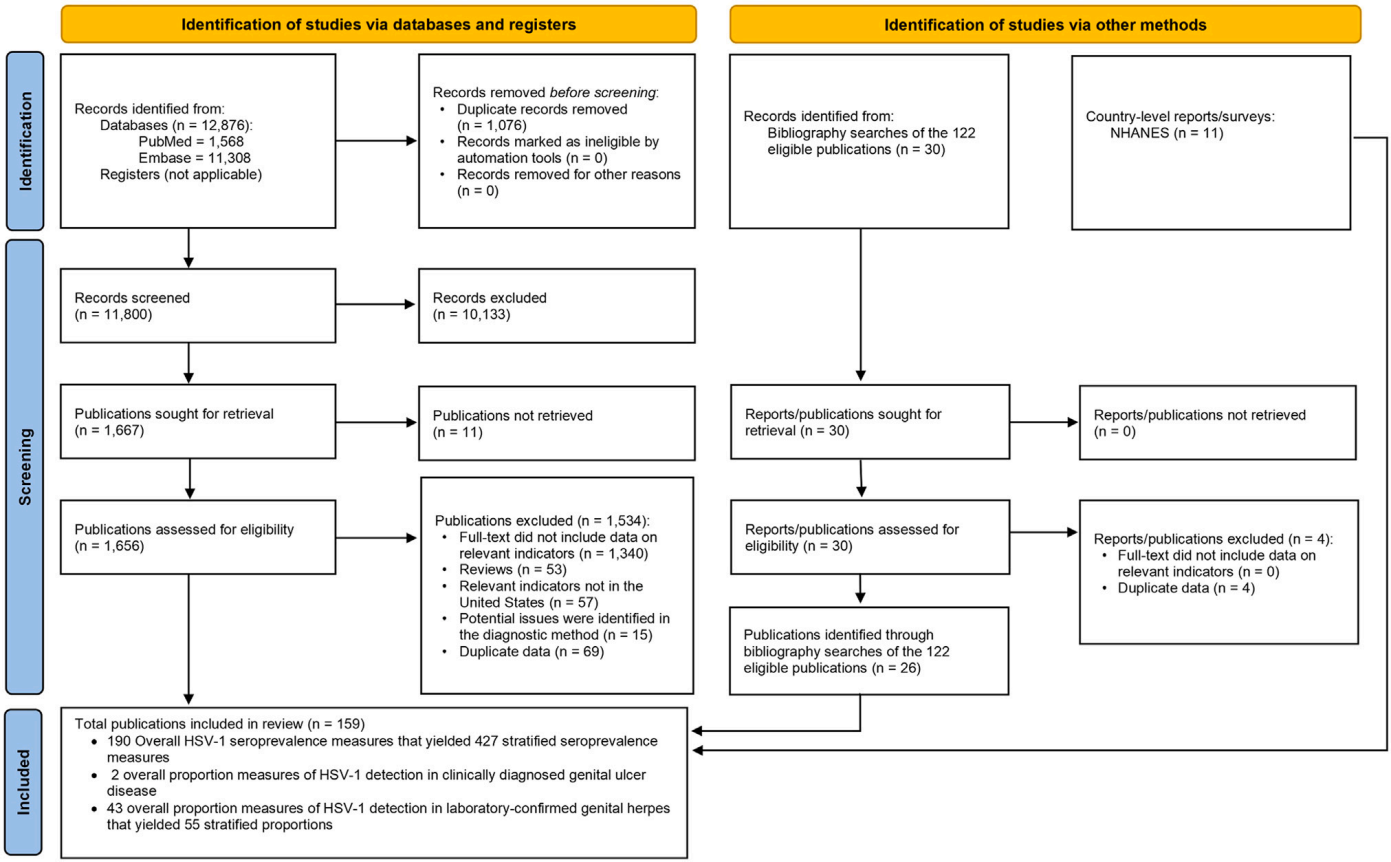
Flowchart of article selection for the systematic review of HSV-1 infection in the United States, according to PRISMA guidelines^27^

Figure 1 depicts the screening and study selection processes. Initially, the search yielded 12,876 records, with 1,568 from PubMed and 11,308 from Embase. A total of 122 of these publications were found to be relevant. Twenty-six more publications that met the inclusion criteria were identified through bibliographic screening of relevant articles and reviews. Eleven National Health and Nutrition Examination Survey (NHANES) datasets were retrieved and analyzed for seroprevalence data.^26^ In total, 159 records/publications were included, and relevant data were extracted from them.

The extracted HSV-1 measures comprised 190 overall seroprevalence measures (including 427 stratified measures), 2 overall proportions of HSV-1 detection in GUD (with no stratified measures for these 2 overall proportions), and 43 overall proportions of HSV-1 detection in genital herpes (including 55 stratified measures).

Cohen’s kappa statistic was estimated at 0.98 (95% confidence interval (CI), 0.96–0.99) for the total number of tested subjects and at 0.98 (95% CI, 0.95–1.00) for the number of positive HSV-1 cases, indicating excellent agreement between the two reviewers on seroprevalence measures. For proportion measures of HSV-1 detection in genital herpes cases, kappa was estimated at 0.95 (95% CI, 0.89–1.00) for the total number of genital herpes cases and at 0.97 (95% CI, 0.91–1.00) for the number of cases caused by HSV-1, also indicating excellent agreement.

### Herpes simplex virus type 1 seroprevalence overview

Table S1 lists the 190 overall HSV-1 seroprevalence measures and associated information, spanning records/publications from as early as 1971 to the most recent one in 2022. The majority of the studies (*n* = 128; 67.4%) were published after 2000. Among the included studies, a substantial portion (*n* = 115; 60.5%) employed convenience sampling instead of probability-based sampling methods. The stratified seroprevalence measures (*n* = 427) exhibited variation across populations and subpopulations, with a median seroprevalence of 60.5% (as detailed in Table 1). Table 1 provides an overview of the ranges and medians of stratified HSV-1 seroprevalence measures, classified by population type, age group and bracket, sex, year(s) of data collection, and year of publication.

**Table 1:** Pooled mean estimates for HSV-1 seroprevalence in the United States

Populations	Outcome measures	Samples	HSV-1 seroprevalence		Pooled mean HSV-1 seroprevalence	Heterogeneity measures		
	Total n	Total N	Range	Median	Mean (95% CI)	Q^a^ (*p*-value)	I^2^^b^ (%) (95% CI)	Prediction^c^ Interval (%)
**Healthy general populations**								

Children	29	6,000	17.6–87.5	32.3	38.0 (30.9–45.4)	376.7 (*p* < 0.001)	92.6 (90.4–94.2)	5.7–78.1
Adults	291	176,650	24.2–100	61.9	63.5 (61.3–65.7)	12,888.3 (*p* < 0.001)	97.7 (97.6–97.9)	26.3–93.3
Age-mixed	11	2,389	20.0–64.0	54.2	49.4 (40.9–58.0)	235.7 (*p* < 0.001)	95.8 (93.9–97.0)	19.2–79.8
All healthy general populations	331	185,039	17.6–100	60.0	61.0 (58.7–63.2)	15,252.0 (*p* < 0.001)	97.8 (97.7–97.9)	21.8–93.2

**Clinical populations**								

Clinical adults	52	17,566	30.0–91.6	62.2	63.5 (58.8–68.1)	1,196.0 (*p* < 0.001)	95.7 (95.0–96.4)	29.7–91.2
Clinical age-mixed	8	1,775	26.4–92.9	45.5	50.9 (35.4–66.4)	235.3 (*p* < 0.001)	97.0 (95.6–98.0)	4.4–96.4
All clinical populations	60	19,341	26.4–92.9	61.6	61.8 (57.1–66.4)	1,658.6 (*p* < 0.001)	96.4 (95.9–96.9)	26.2–91.5

**Other populations**								

HIV positive patients	14	5,006	55.7–76.0	67.2	67.9 (63.6–72.0)	70.8 (*p* < 0.001)	81.6 (70.3–88.7)	52.3–81.7
Men who have sex with men	8	1,355	41.0–64.7	53.4	55.7 (51.3–60.0)	13.2 (*p* = 0.067)	47.0 (0.0–76.4)	44.4–66.7
Mixed populations at risk	3	2,907	64.0–77.9	72.7	72.0 (60.3–82.3)	53.0 (*p* < 0.001)	96.2 (92.0–98.2)	0.0–100
Partners of genital herpes patients	4	729	55.4–63.2	58.0	58.9 (50.9–62.5)	0.9 (*p* = 0.831)	0.0 (0.0–84.7)	50.9–66.7
Population exposed to sexual abuse	3	375	45.6–85.3	74.2	68.7 (43.1–89.5)	42.1 (*p* < 0.001)	95.3 (89.4–89.5)	0.0–100
Women who have sex with women	4	392	39.0–62.0	45.0	47.4 (37.2–57.7)	12.2 (*p* = 0.007)	75.4 (31.9–91.1)	8.9–87.8

**Specific sub-populations of epidemiological relevance**								

Healthy pregnant women^d^	11	67,936	41.4–69.1	58.2	59.9 (55.1–64.7)	609.3 (*p* < 0.001)	98.4 (97.9–98.7)	40.9–77.5
HIV positive females^e^	6	4,171	64.0–76.0	70.8	73.4 (70.8–76.0)	11.3 (*p* = 0.046)	55.6 (66.3–82.2)	66.3–80.0
HIV positive males^e^	4	237	56.0–75.9	68.1	68.3 (58.4–77.4)	7.26 (*p* = 0.064)	58.7 (0.0–86.2)	28.1–97.0
HIV positive sex-mixed^e^	4	598	55.7–68.4	61.8	61.4 (55.1–67.5)	6.7 (*p* = 0.083)	55.0 (0.0–85.1)	36.7–83.4
HSV-2 positive patients^f^	3	676	38.0–62.3	62.1	53.2 (36.4–69.6)	28.0 (*p* < 0.001)	92.9 (82.4–97.1)	0.0–100
STD clinic attendees^f^	22	11,406	31.5–91.1	61.5	60.2 (53.8–66.5)	466.0 (*p* < 0.001)	95.5 (94.2–96.5)	29.1–87.4

**Sex**								

Females	188	143,121	18.0–100	61.5	61.7 (59.0–64.3)	7,831.7 (*p* < 0.001)	97.6 (97.4–97.8)	25.9–91.4
Males	145	33,087	17.6–100	58.8	59.7 (56.1–63.2)	4,544.7 (*p* < 0.001)	96.8 (96.5–97.1)	19.1–93.7
Mixed sexes	94	38,936	20.0–95.1	61.1	62.3 (58.4–66.1)	4,779.2 (*p* < 0.001)	98.1 (97.9–98.2)	25.4–92.5

**Age group**								

<10 years	5	1,279	24.6–59.0	27.9	32.5 (22.0–44.0)	29.8 (*p* < 0.001)	86.8 (70.8–93.8)	2.1–76.5
10-19 years	54	23,798	17.6–87.5	35.7	39.5 (35.1–44.0)	972.6 (*p* < 0.001)	94.6 (93.6–95.4)	11.2–72.3
20-29 years	75	37,807	26.7–100	53.2	56.6 (52.7–60.4)	1,190.7 (*p* < 0.001)	93.8 (92.8–94.6)	24.4–86.0
30-39 years	49	12,038	41.7–91.2	63.1	63.5 (60.9–66.0)	302.1 (*p* < 0.001)	84.1 (79.7–87.5)	46.0–79.3
40-49 years	45	9,985	53.8–95.9	65.5	68.0 (65.2–70.7)	274.1 (*p* < 0.001)	83.9 (79.3–87.6)	49.5–84.0
≥50 years	34	7,166	57.3–100	89.8	88.3 (85.2–91.2)	662.0 (*p* < 0.001)	95.0 (93.9–95.9)	66.5–99.6
Mixed	165	123,071	20.0–100	62.0	61.8 (59.2–64.3)	6,252.1 (*p* < 0.001)	97.4 (97.2–97.6)	29.6–89.2

**Year of publication category**^g^								

<2000	134	46,690	30.0–100	71.1	72.3 (69.3–75.2)	4,250.3 (*p* < 0.001)	96.9 (96.6–97.1)	36.1–96.9
2000–2009	155	85,186	20.0–93.8	58.3	56.9 (54.2–59.6)	6,525.1 (*p* < 0.001)	97.6 (97.5–97.8)	24.7–86.2
≥2010	138	83,268	17.6–100	54.4	54.6 (51.3–57.8)	6,387.7 (*p* < 0.001)	97.9 (97.9–98.0)	19.4–87.3

**Year of data collection category**^g^								

<1995	144	109,388	30.0–100	71.1	72.1 (69.3–74.8)	5,037.2 (*p* < 0.001)	97.2 (96.9–97.4)	36.9–96.4
1995–2004	140	64,309	24.6–100	57.0	58.0 (55.0–61.0)	5,006.7 (*p* < 0.001)	97.2 (97.0–97.4)	23.9–88.4
≥2005	143	41,447	17.6–95.1	54.2	52.6 (49.7–55.5)	4,749.1 (*p* < 0.001)	97.0 (96.7–97.3)	20.1–83.9

**Age bracket**								

All children	31	6,314	17.6–87.5	32.7	39.3 (32.2–46.6)	407.6 (*p* < 0.001)	92.6 (90.6–94.2)	6.2–79.5
All adults	376	204,605	24.2–100	62.0	63.3 (61.5–65.2)	14,888.0 (*p* < 0.001)	97.5 (97.4–97.6)	27.8–92.2
All age-mixed	20	4,225	20.0–92.9	52.8	51.8 (43.3–60.2)	513.5 (*p* < 0.001)	96.3 (95.3–97.1)	15.0–87.5
**All studies**	**427**	**215,144**	**17.6–100**	**60.5**	**61.1 (59.2–63.0)**	**17,810.0 (*p* < 0.001)**	**97.6 (97.5–97.7)**	**23.7–92.3**

CI, confidence interval; HSV-1, herpes simplex virus type 1; HSV-2, herpes simplex virus type 2; HIV, human immunodeficiency virus; STD, sexually transmitted disease.

a Q: The Cochran’s Q statistic is a measure assessing the existence of heterogeneity in pooled outcome measures, here HSV-1 seroprevalence.

b I^2^: A measure assessing the magnitude of between-study variation that is due to true differences in HSV-1 seroprevalence across studies rather than sampling variation.

c Prediction interval: A measure quantifying the distribution (95% interval) of true HSV-1 seroprevalence around the estimated pooled mean.

d This population was included as part of the healthy general population; however, a separate analysis was performed for public health relevance.

e This population was included as part of the HIV positive patients; however, a separate analysis based on sex was performed for epidemiological relevance.

f This population was included as part of the clinical populations; however, a separate analysis was performed for epidemiological relevance.

g The categories were determined based on the median time observed between the year of publication and the year of data collection, which was approximately 3 years. To create distinct brackets, this interval was approximated to 5 years, to have 5-year intervals.

### Precision, risk of bias, and publication bias assessments

After assessing the diagnostic methods, fifteen publications were excluded due to potential validity issues in diagnostic assays (Figure 1). The precision and risk of bias (ROB) assessments of the 190 seroprevalence studies are summarized in Table S2. Among these studies, 164 (86.3%) demonstrated high precision, while 74 (38.9%) exhibited low ROB in the sampling method domain, and 27 (14.2%) showed low ROB in the response rate domain. Conversely, 26 studies (13.7%) had low precision, 116 studies (61.1%) had high ROB in the sampling method domain, and 45 (23.7%) had high ROB in the response rate domain.

Twenty-two studies (11.6%) were identified as having low ROB in both quality domains, while the number of studies with high ROB in both quality domains was 9 (4.7%). The ROB assessment for the response rate domain was categorized as "unclear" for 118 studies (62.1%). Notably, in the meta-regressions for HSV-1 seroprevalence (as presented in Table 2), both study precision and response rate showed a statistically significant association with HSV-1 seroprevalence.

**Table 2:** Univariable and multivariable meta-regression analyses for HSV-1 seroprevalence in the United States using year of data collection as a linear term

			Outcome measures	Samples	Univariable analysis				Multivariable analysis			
									Model 1^a^		Model 2^b^	
			Total n	Total N	*RR* (95%CI)	*p*-value	LR test *p*-value	Adjusted R^2^ (%)	*ARR* (95%CI)	*p*-value	*ARR* (95%CI)	*p*-value
**Population Characteristics**	**Age bracket**	**Children**	31	6,314	1.00	–	<0.001	13.35	–	–	1.00	–
		**Adults**	376	204,605	1.61 (1.43–1.81)	<0.001			–	–	1.39 (1.25–1.56)	<0.001
		**Age-mixed**	20	4,225	1.32 (1.10–1.58)	0.002			–	–	1.16 (0.98–1.37)	0.080
	**Age group**	<10	5	1,279	1.00	–	<0.001	43.44	1.00	–	–	–
		10–19	54	23,798	1.21 (0.96–1.54)	0.112			1.29 (1.04–1.60)	0.020	–	–
		20–29	75	37,807	1.73 (1.36–2.19)	<0.001			1.77 (1.42–2.20)	<0.001	–	–
		30–39	49	12,038	1.98 (1.56–2.52)	<0.001			2.12 (1.70–2.65)	<0.001	–	–
		40–49	45	9,985	2.12 (1.67–2.70)	<0.001			2.29 (1.83–2.87)	<0.001	–	–
		≥50	34	7,166	2.74 (2.15–3.49)	<0.001			2.47 (1.97–3.10)	<0.001	–	–
		Mixed	165	123,071	1.89 (1.50–2.39)	<0.001			1.81 (1.46–2.24)	<0.001	–	–
	**Sex**	Female	188	143,121	1.00	–	0.310^c^	0.06	1.00	–	1.00	–
		Male	145	33,087	0.95 (0.89–1.02)	0.183			0.96 (0.91–1.01)	0. 094	0.96 (0.91–1.02)	0.208
		Mixed	94	38,936	1.01 (0.93–1.09)	0.843			1.00 (0.94–1.07)	0.879	1.04 (0.97–1.12)	0.236
	**Population type**	Healthy	331	185,039	1.00	–	0.568	0.29	–	–	–	–
		Clinical	60	19,341	1.03 (0.94–1.13)	0.508			–	–	–	–
		Other	36	10,764	1.05 (0.94–1.18)	0.366			–	–	–	–
**Study methodology characteristics**	**Assay type**	Western blot	117	136,170	1.00	–	0.265	0.14	–	–	–	–
		ELISA	303	77,725	0.95 (0.89–1.02)	0.149			–	–	–	–
		Others	7	1,249	0.87 (0.67–1.13)	0.305			–	–	–	–
	**Sample size**^d^	<100	27	1,385	1.00	–	0.075	0.26	1.00	–	1.00	–
		≥100	400	213,759	1.13 (0.99–1.30)	0.075			1.19 (1.07–1.32)	0.002	1.21 (1.08–1.37)	0.002
	**Sampling method**	Probability-based	253	69,851	1.00	–	0.165	0.22	–	–	–	–
		Non-probability-based	174	145,293	1.05 (0.98–1.12)	0.152			–	–	–	–
	**Response rate**	≥80	58	25,507	1.00	–	<0.001	11.90	1.00	–	1.00	–
		<80	193	58,015	1.39 (1.27–1.53)	<0.001			1.02 (0.94–1.11)	0.639	1.27 (1.17–1.38)	<0.001
		Unclear	176	131,622	1.33 (1.21–1.46)	<0.001			1.12 (1.03–1.23)	0.011	1.24 (1.13–1.36)	<0.001
**Year of data collection as a linear term**			427	215,144	0.99 (0.98–0.99)	<0.001	<0.001	20.26	0.99 (0.99–0.99)	<0.001	0.99 (0.98–0.99)	<0.001

*ARR*, adjusted risk ratio; CI, confidence interval; HSV-1, herpes simplex virus type 1; *RR*, risk ratio.

a Variance explained by the final multivariable model 1 (adjusted *R*^*2*^) = 57.04%.

b Variance explained by the final multivariable model 2 (adjusted *R*^*2*^) = 38.98%.

c Although sex variable did not have a statistically significant association with the outcome in the univariable analysis (*p*-value>0.1), it was included in the multivariable analysis because of its epidemiological relevance.

d Sample size denotes the sample size of the study population found in the original publication.

Publication bias assessment is summarized in Table S3, with the Doi plots included in Figure S1. While there was no evidence of publication bias in some meta-analyses, others demonstrated asymmetrical Doi plots and Luis Furuya-Kanamori (LFK) index values exceeding ±1, indicating the presence of publication bias.

### Pooled mean estimates for herpes simplex virus type 1 seroprevalence

The pooled mean HSV-1 seroprevalence was estimated to be 38.0% (95% CI: 30.9–45.4) in healthy children from the general population, 63.5% (95% CI: 61.3–65.7) in healthy adults from the general population, and also 63.5% (95% CI: 58.8–68.1) in clinical adult populations (Table 1).

Forest plots in Figures S2 and S3 illustrate the results of the meta-analyses for each population group. Most meta-analyses showed significant heterogeneity (*p*-value<0.001), primarily due to true variations in HSV-1 seroprevalence across studies rather than sampling variation (I^2^>50%). Wide prediction intervals confirmed substantial variability in HSV-1 seroprevalence across the studies.

### Sources of between-study heterogeneity and predictors of HSV-1 seroprevalence

The identified heterogeneity in the meta-analyses was investigated using univariable and multivariable meta-regression analyses to explain the factors behind the observed heterogeneity. Tables 2 and S4 present the results of these analyses for HSV-1 seroprevalence. Two multivariable models were employed: one with the year of data collection as a linear term (Table 2) and another with the year of data collection as a categorical variable (Table S4). To address collinearity between variables, additional analyses were conducted, replacing age group with age bracket (children versus adults) as the age variable in both Tables 2 and S4, and using the year of publication instead of the year of data collection as the time variable (Table S5). Both the primary and additional analyses yielded consistent outcomes.

The primary analysis, utilizing the year of data collection as a linear term, accounted for 57% of the variation in seroprevalence across the studies (Table 2). Seroprevalence increased progressively with age, with age being the most important factor contributing to the variability in seroprevalence measures. Age alone explained 43% of the variation in seroprevalence. There was no evidence of seroprevalence differences based on sex or between healthy general populations and clinical populations. The results strongly supported a decline with time in seroprevalence, occurring at a relative rate of 1% per year.

Regarding the impact of study methods on seroprevalence, there was no evidence of variation in seroprevalence based on assay type or sampling method (Table 2). There was also no consistent evidence for variation in seroprevalence based on the study response rate, and the effect size was small. However, there was evidence of an effect based on sample size, as studies with a sample size of ≥100 reported 19% higher seroprevalence.

### Herpes simplex virus type 1 detection in clinically diagnosed genital ulcer disease and in laboratory-confirmed genital herpes

Table S6 presents the overall proportions of HSV-1 detection in GUD and in genital herpes, while Table 3 provides a summary of their stratified measures. Among GUD cases (*n* = 2), the pooled mean proportion of HSV-1 detection was 18.0% (95% CI: 10.4–27.2), while in genital herpes cases (*n* = 55), the pooled mean proportion of HSV-1 detection was 15.4% (95% CI: 10.8–20.6).

**Table 3:** Pooled mean proportions of HSV-1 virus detection in clinically diagnosed genital ulcer disease and in laboratory-confirmed genital herpes in the United States

Population type	Outcome measures	Samples	Proportion of HSV-1 detection		Pooled proportion of HSV-1 detection	Heterogeneity measures		
	Total n	Total N	Range	Median	Mean (95% CI)	Q^a^ (*p*-value)	I^2^^b^ (%) (95% CI)	Prediction^c^ Interval (%)
**Patients with clinically diagnosed GUD**								

**All patients with GUD**	**2**^d^	**699**	**14.3–22.8**	**18.5**	**18.0 (10.4–27.2)**	–	–	–

**Patients with laboratory-confirmed genital herpes**								

**Sex**								
Females	14	2,749	0.0–78.3	30.6	24.2 (12.6–38.1)	474.9 (*p* < 0.001)	97.3 (96.4–97.9)	0.0–82.4
Males	11	3,003	0.0–67.9	27.3	20.1 (8.8–34.2)	450.0 (*p* < 0.001)	97.8 (97.0–98.3)	0.0–77.2
Mixed	30	6,058	0.0–52.4	9.9	10.5 (6.3–15.7)	508.6 (*p* < 0.001)	94.3 (92.8–95.5)	0.0–45.9
**Genital herpes episode status**								
Primary genital herpes	21	6,008	2.8–54.1	27.3	24.4 (18.5–30.9)	336.8 (*p* < 0.001)	94.1 (92.1–95.5)	2.9–56.6
Recurrent genital herpes	15	2,207	0.0–10.0	0.0	1.6 (0.4–3.4)	59.6 (*p* < 0.001)	76.5 (61.4–85.7)	0.0–10.5
Unclear genital herpes episode	19	3,595	3.2–78.3	17.4	22.6 (13.5–33.2)	467.1 (*p* < 0.001)	96.1 (95.0–97.0)	0.0–73.9
**Year of data collection category**^e^								
<2000	39	7,294	0.0–54.1	10.0	11.9 (7.7–16.8)	844.9 (*p* < 0.001)	95.5 (94.6–96.3)	0.0–49.6
≥2000	16	4,516	0.0–78.3	28.8	25.3 (14.1–38.4)	461.8 (*p* < 0.001)	96.8 (95.8–97.5)	0.0–82.8
**Year of publication category**^e^								
<2005	37	7,058	0.0–54.1	9.9	10.9 (6.8–15.8)	785.7 (*p* < 0.001)	95.4 (94.4–96.2)	0.0–47.5
≥2005	18	4,752	0.0–78.3	29.2	26.2 (16.0–37.8)	463.1 (*p* < 0.001)	96.3 (95.2–97.2)	0.0–79.9
**All patients with genital herpes**	**55**	**11,810**	**0.0–78.3**	**12.5**	**15.4 (10.8–20.6)**	**1,820.8 (*p* < 0.001)**	**97.0 (96.6–97.4)**	**0.0–60.7**

CI, Confidence interval; GUD, Genital ulcer disease; HSV-1, Herpes simplex virus type 1.

a Q: The Cochran’s Q statistic is a measure assessing the existence of heterogeneity in pooled outcome measures, here proportions of HSV-1 virus detection.

b I^2^: A measure assessing the magnitude of between-study variation that is due to true differences in proportions of HSV-1 virus detection across studies rather than sampling variation.

c Prediction interval: A measure quantifying the distribution (95% interval) of true proportions of HSV-1 virus detection around the estimated pooled mean.

d No meta-analysis was done as number of studies was <3.

e The categories were determined based on the median time observed between the year of publication and the year of data collection, which was approximately 3 years. To create distinct brackets, this interval was approximated to 5 years, to have 5-year intervals.

All the meta-analyses displayed evidence of heterogeneity (*p*-value<0.001) and showed wide prediction intervals (Table 3). The heterogeneity observed was primarily due to true differences in these proportions rather than being attributed to sampling variation (I^2^>50%). For visual representation, the forest plot for the genital herpes meta-analysis is provided in Figure S4.

### Sources of between-study heterogeneity and predictors of herpes simplex virus type 1 detection in genital herpes

Table 4 presents the results of both univariable and multivariable meta-regression analyses for the proportion of HSV-1 detection in genital herpes. Two multivariable models were utilized: one with the year of data collection as a categorical variable and another with the year of data collection as a linear term. Both models yielded consistent results.

**Table 4:** Univariable and multivariable meta-regression analyses for HSV-1 virus detection in laboratory-confirmed genital herpes in the United States

	Outcome measures	Samples	Univariable analysis				Multivariable analysis			
							Model 1^a^		Model 2^b^	
	Total n	Total N	*RR* (95%CI)	*p*-value	LR test *p*-value	Adjusted R^2^ (%)	*ARR* (95%CI)	*p*-value	*ARR* (95%CI)	*p*-value
**Sex**										

Females	14	2,749	1.00	–	0.138^c^	5.24	1.00	1.00	1.00	–
Males	11	3,003	0.87 (0.40–1.89)	0.720			0.84 (0.46–1.55)	0.577	0.82 (0.44–1.52)	0.524
Mixed	30	6,058	0.55 (0.29–1.04)	0.065			0.60 (0.37–0.99)	0.045	0.64 (0.39–1.05)	0.077

**Genital herpes episode status**										

First-episode genital herpes	21	6,008	1.00	–	<0.001	40.07	1.00	1.00	1.00	–
Recurrent genital herpes	15	2,207	0.19 (0.10–0.37)	<0.001			0.19 (0.10–0.36)	<0.001	0.17 (0.09–0.32)	<0.001
Unspecified status	19	3,595	0.87 (0.54–1.40)	0.556			0.68 (0.42–1.10)	0.113	0.68 (0.42–1.10)	0.117

**Year of data collection (category)**										

<2000	39	7,294	1.00	–	0.07	6.92	1.00	–	–	–
≥2000	16	4,516	1.69 (0.96–2.98)	0.070			1.58 (0.97–2.57)	0.065	–	–
**Year of data collection as a linear term**	55	11,810	1.01 (0.99–1.04)	0.198	0.198	1.46	–	–	1.02 (1.00–1.04)	0.054

*ARR*, adjusted risk ratio; CI, confidence interval; HSV-1, herpes simplex virus type 1; *RR*, risk ratio.

a Variance explained by the final multivariable model (adjusted *R*^*2*^) = 49.46%.

b Variance explained by the final multivariable model (adjusted *R*^*2*^) = 48.73%.

c Although the sex variable did not have a statistically significant association with the outcome in the univariable analysis (*p*-value>0.1), it was included in the multivariable analysis because of its epidemiological relevance.

The model employing the year of data collection as a linear term explained 49% of the variation in the proportion of HSV-1 detection. Among the predictors, genital herpes status emerged as the most important factor, alone accounting for 40% of the variation in this proportion. Specifically, recurrent genital herpes displayed a 0.17-fold (95% CI: 0.09–0.32) lower proportion of HSV-1 detection compared to first-episode genital herpes. The results also supported a tendency for an increasing proportion over time, at a relative rate of 2% per year (*p*-value = 0.054).

## Discussion

The results indicate a 1% annual decline in seroprevalence over recent decades, with both children and adults exhibiting substantially lower seroprevalence compared to previous decades^11^^,^^12^^,^^13^^,^^28^^,^^29^^,^^30^^,^^31^^,^^32^ and to that observed in other world regions.^19^^,^^20^^,^^21^^,^^22^ Conversely, the detection of HSV-1 in genital herpes cases has increased by 2% annually. Collectively, these findings suggest a gradual transition in the mode of HSV-1 acquisition, characterized by a decline in oral acquisition—often occurring in childhood^7^^,^^8^—and a concerning rise in genital acquisition.

HSV-1 seroprevalence is substantially below its historical level of nearly universal childhood infection, which is still seen in most parts of the world.^19^^,^^20^^,^^21^^,^^22^ However, it is comparable to the levels observed in other Western countries, such as in Europe and Canada.^23^^,^^24^ These findings, for both the levels and trends, corroborate analyses of NHANES data over the decades^11^^,^^28^^,^^29^^,^^30^^,^^31^ results of observational cohorts,^12^^,^^13^^,^^32^ and a mathematical modeling study investigating HSV-1 oral and genital transmissions and seroprevalence trends.^14^

The declining seroprevalence, possibly linked to the general decrease in both family size and school crowding, along with improved hygiene,^12^^,^^28^^,^^33^ supports the influence of living conditions during childhood on the risk of infection.^28^ This decrease in seroprevalence can be viewed as a positive development, indicating the lower rates of morbidities associated with the oral acquisition of the infection. However, this decline is also accompanied by increasing rates of genital acquisition, as many adolescents are reaching sexual debut uninfected and susceptible to genital acquisition through mostly oral-genital sex.^14^ This type of acquisition not only leads to genital herpes morbidity but also contributes to a range of detrimental sexual, social, and psychological outcomes, including negative impacts on sexual relations, quality of life, social stigmatization, and mental well-being, such as depression, anxiety, and low self-esteem.^34^^,^^35^^,^^36^^,^^37^

The growing trend in HSV-1-caused genital herpes is similar to the trends observed in Europe,^23^ Australia,^25^ and Canada,^24^ and is partly due to the concurrent decline in HSV-2 seroprevalence.^38^ However, the primary driver of the growing trend is the decreasing HSV-1 seroprevalence, as the absolute decline in HSV-1 seroprevalence is much larger than that in HSV-2 seroprevalence.^14^^,^^38^ The progress in reducing oral acquisition has led to the emergence of genital acquisition, which historically played a limited role in the infection’s epidemiology. Consequently, this transition has resulted in new forms of disease burden for this infection and its increasing recognition as a sexually transmitted infection.

HSV-1 detection in first-episode genital herpes was six times higher compared to recurrent genital herpes. This finding supports clinical observations indicating that HSV-1 reactivation in the genital tract tends to occur for a shorter duration than HSV-2.^6^^,^^37^

Age emerged as the most influential factor in explaining the variation in observed seroprevalence, while other factors such as clinical condition showed no effect on seroprevalence. This finding confirms the strong role of age in exposure, particularly for the oral mode of transmission, consistent with observations in other regions.^19^^,^^20^^,^^21^^,^^22^^,^^23^^,^^24^^,^^25^ The results showed that there are no differences in HSV-1 seroprevalence by sex, which is also consistent with observations from other populations and regions.^19^^,^^20^^,^^21^^,^^22^

Interestingly, the pooled mean seroprevalence of approximately 60% was similar to the average seroprevalence estimated in NHANES surveys over the same duration, which was also around 60%,^11^^,^^26^^,^^28^^,^^29^^,^^30^^,^^31^ despite the fact that a large proportion of studies included in the meta-analysis relied on convenience sampling rather than probability-based methods. The sampling method did not influence seroprevalence, as confirmed by the meta-regression analysis of seroprevalence measures (Table 2). These findings demonstrate how this infection is truly a general population infection, with age being the most influential determinant.

The findings underscore the common occurrence of oral and genital HSV-1 infections, highlighting the need for heightened attention in medical practice and public health initiatives. The observed increase in genital HSV-1, in contrast to genital HSV-2,^38^ poses distinct challenges in sexual health contexts, impacting diagnosis, treatment, management, and counseling. Notably, genital HSV-1 demonstrates fewer recurrences than HSV-2,^6^ as demonstrated also in this study, but can still significantly distress patients during outbreaks and pose serious risks during childbirth, such as more severe or even fatal neonatal herpes compared to HSV-2.^9^^,^^10^^,^^37^ Importantly, incident HSV-1 appears to be more easily transmitted to neonates than HSV-2.^39^

Accurate differentiation between HSV-1 and HSV-2 is critical for managing cases effectively, including conveying the reduced transmission risk in sexual partnership and potentially milder prognosis of genital HSV-1.^6^ Clinicians must remain vigilant when evaluating patients with GUD or suspected genital herpes, ensuring appropriate testing protocols are employed for precise diagnosis, treatment, and management. Moreover, the findings highlight the need for tailored educational campaigns to correct misconceptions and accurately inform the public about herpes infections. The study’s findings also stress the importance of ongoing surveillance and the development of preventive measures, such as HSV-1 vaccines,^17^^,^^18^ to address the shifting epidemiology of HSV-1 and mitigate its impact on public health.

In conclusion, the different results of this study are consistent with HSV-1 epidemiology in the United States undergoing a transition, moving away from the historical pattern of acquiring the infection primarily during childhood through the oral route. Most adolescents are reaching sexual debut unexposed to this infection, and thus at risk of genital acquisition. As a consequence, seroprevalence is steadily declining by 1% per year, yet this decline is paradoxically contributing to an increasing trend in HSV-1 genital herpes at a rate of 2% per year. These findings underscore the importance of continuous disease surveillance and monitoring of HSV-1 seroprevalence and genital herpes etiology and provide compelling support for the development and deployment of an HSV-1 vaccine, alongside other public health interventions, to effectively mitigate the disease burden associated with this infection.

### Limitations of the study

This study had limitations. The systematic search utilized the PubMed and Embase databases, excluding others such as Scopus and Web of Science. However, Scopus and Web of Science generally draw from the same sources as PubMed and Embase. Given the study’s focus on the United States, PubMed is especially critical as it comprehensively encompasses published biomedical research within the country. A substantial volume of evidence was identified, enabling multiple analyses and the generation of diverse inferences. Consequently, the inclusion of a few potentially missed studies is unlikely to significantly affect the results derived from the large number of studies already included.

Included studies exhibited variations in sample size, sampling method, and response rate, as well as the use of different diagnostic assays. However, no significant effect was found on seroprevalence for any of the study methods, except for a minor effect related to sample size. Hence, the variability in study methods may not have impacted the study’s findings. Availability of data was specifically limited for HSV-1 detection in GUD. There was evidence of publication bias in some meta-analyses; however, this was primarily observed among specific populations rather than the general population.

Despite the observed heterogeneity in the included studies' measures, approximately half of this variation was subsequently explained through the meta-regression analyses, considering epidemiological factors such as age and time trend. This finding indicates that the observed heterogeneity is mostly attributed to the natural variation that exists in HSV-1 epidemiology. A key strength of this study is the extensive volume of HSV-1 seroprevalence and genital herpes data, surpassing that found in other countries,^19^^,^^20^^,^^21^^,^^22^^,^^23^^,^^24^^,^^25^ which facilitated an array of analyses and resulted in influential insights.

## STAR★Methods

### Key resources table

**Table:** 

REAGENT or RESOURCE	SOURCE	IDENTIFIER
**Deposited data**		

Analysis codes files, including Stata Do files and R Scripts.	Zenodo	https://doi.org/10.5281/zenodo.12739913

**Other**		

Data published/compiled from the literature.	PubMed database	www.ncbi.nlm.nih.gov/pubmed
Data published/compiled from the literature.	Embase database	www.ovid.com
Data published/compiled from the literature.	NHANES reports	NHANES Questionnaires, Datasets, and Related Documentation (cdc.gov)

**Software and algorithms**		

Stata/SE version 17, utilizing "metareg" package - Stata: Statistical software for data science.	StataCorp LLC, USA	https://www.stata.com/
R version 4.0.4, utilizing the "meta" package - The R Project for Statistical Computing.	R Core team, R Foundation for Statistical Computing, AT	https://www.r-project.org/
EndNote version X9 - EndNote reference manager.	Clarivate Plc, USA, UK	https://endnote.com/

### Resource availability

#### Lead contact

Further information and requests for resources should be directed to and will be fulfilled by the corresponding author, Laith J. Abu-Raddad (lja2002@qatar-med.cornell.edu).

#### Materials availability

This study did not generate new unique reagents.

#### Data and code availability

• This paper analyses existing, publicly available data. All data were extracted from published articles and databases and are listed in Tables S1 and S6.
• All original code has been deposited at Zenodo data repository and is publicly available as of the date of publication. DOIs are listed in the key resources table.
• Any additional information required to re-analyse the data reported in this paper is available from the lead author.

### Experimental model and study participant details

This is not applicable to our study, as it is a systematic review and does not involve experimental or human subjects research.

### Method detail

The methods, encompassing the search for relevant publications, study selection based on eligibility criteria, data extraction procedures, and subsequent data evaluations and analyses, are described within the STAR methods section of the main text and the supplemental information. This detailed methodological description is a standard aspect of conducting a systematic review in accordance with established reporting guidelines and the recommended structure for systematic reviews.

#### Methods

The approach utilized in this research was based on a set of previously published systematic reviews investigating the epidemiology of HSV-1 and HSV-2 infections in various regions and countries.^19^^,^^20^^,^^21^^,^^22^^,^^23^^,^^24^^,^^25^^,^^40^^,^^41^^,^^42^^,^^43^^,^^44^^,^^45^^,^^46^ As a result, this study’s protocol was not registered with PROSPERO. A description of the methodology is provided in Methods S1, and a summary of the methodology is presented below.

##### Data sources and search strategy

This systematic review followed the guidance of the Cochrane Collaboration Handbook.^47^ The findings were reported in accordance with the Preferred Reporting Items for Systematic Reviews and Meta-analyses (PRISMA) guidelines.^27^^,^^48^ The PRISMA checklist can be found in the Table S7. A comprehensive search was conducted on PubMed and Embase databases until April 10, 2023, using exploded Mesh/Emtree terms, free text terms, and broad search criteria, without imposing any restrictions on time or language. The search strategies can be found in Table S8. Non-English articles were translated into English.

Results from rounds of NHANES,^26^ a regularly conducted, nationally representative, probability-based survey following standardized analytical and laboratory procedures, were incorporated. A total of eleven consecutive surveys ('waves' or 'rounds') conducted between 1976 and 2016 were analyzed.^26^ The extraction and analysis of seroprevalence data followed NHANES' standardized 'survey methods and analytic guidelines'.^49^

##### Study selection and eligibility criteria

The methodology for screening and selecting studies is outlined in Methods S1. Using the reference manager Endnote (Thomson Reuters, USA), citations were imported from PubMed and Embase databases, and duplicate citations were removed. Two authors (RA, MH) independently conducted title and abstract screening to identify relevant and potentially relevant publications. Full texts of these publications were then obtained for further evaluation. A bibliography screening of relevant publications and reviews was performed to identify any additional potentially relevant studies.

The inclusion criteria required any publication with a minimum sample size of 10, reporting primary data on HSV-1 seroprevalence, the proportion of HSV-1 detection in clinically diagnosed GUD, and the proportion of HSV-1 (versus HSV-2) detection in laboratory-confirmed genital herpes. The exclusion criteria encompassed case reports, case series, reviews, editorials, commentaries, and qualitative studies. Measures reporting seroprevalence in infants aged less than 6 months were excluded, as their antibodies could be of maternal origin. No other populations were excluded based on the study criteria.

In this article, the terms "record" or "publication" pertain to a document, such as an article or public health report, that contains relevant outcome measures for one or more populations. The terms "study" or "measure" are used to denote a specific outcome measure conducted within a particular population. Duplicate findings from studies were incorporated only once, with preference given to the more detailed publication.

##### Data extraction and data synthesis

Data extraction was performed by RA and double extraction was performed by MH. The variables extracted are specified in Methods S1. Any discrepancies that arose were discussed and resolved through consultation with LJA to reach a consensus. Overall outcome measures (i.e., encompassing the entire sample) and their stratified measures were extracted, with the condition that the sample size in each stratum was ≥10. The stratification hierarchy for seroprevalence measures, as well as for GUD and genital herpes measures, is listed in Methods S1.

Both overall and stratified measures were extracted because the aim of the study was to investigate the natural heterogeneity in HSV-1 epidemiology by categorizing the measures based on key epidemiological factors known to impact the infection’s spread.^19^^,^^20^^,^^21^^,^^22^^,^^23^^,^^24^^,^^25^ Subsequently, meta-regression analyses were conducted on these stratified measures to assess the influence of these epidemiological factors on HSV-1 seroprevalence and the proportion of HSV-1 detection in genital herpes cases. These analyses also sought to explore temporal trends and identify potential sources of variation between studies. This analytical approach provides concrete insights into the infection’s epidemiology by explaining the underlying variations in the available measures.^44^

Inter-reviewer agreement for data extraction was assessed using Cohen’s kappa statistic.^50^ Kappa values below 0.40, between 0.40 and 0.75, and above 0.75 were considered indicative of poor, fair/good, and excellent agreement, respectively.^50^ 95% CIs were calculated.

##### Precision, risk of bias, and publication bias assessments

To address the known limitations of HSV assays,^51^^,^^52^^,^^53^ a quality assessment of the assay used in each relevant study was conducted. For this task, we sought the expertise of Professor Rhoda Ashley-Morrow from the University of Washington—a distinguished authority in HSV serological assays, with three decades of experience investigating and evaluating the validity and reliability of various HSV assays. Information on each assay from each study was shared with Professor Ashley-Morrow, and her expert judgment was utilized to assess their validity and reliability. Only studies with assays deemed valid and reliable were included in this systematic review.

Each study underwent an assessment for precision and ROB by two independent reviewers (RA, MH). These evaluations were informed by the Cochrane approach,^54^ pertinent quality components in prevalence studies,^55^ and a methodology honed through a series of systematic reviews focusing on HSV-1 and HSV-2 seroprevalence.^19^^,^^20^^,^^21^^,^^22^^,^^23^^,^^24^^,^^25^^,^^40^^,^^41^^,^^42^^,^^43^^,^^44^^,^^45^^,^^46^ This methodology, tailored and refined for the research questions in the present study, comprised one component for study precision and two components for ROB.

Other components were not included because they were either inherently satisfied by our study design and inclusion/exclusion criteria, or they pertained to different but more relevant research questions within our study, as detailed in Table S9. For instance, the validity and reliability of the study instrument measuring the parameter of interest were implicitly assessed through the involvement of Professor Ashley-Morrow as described above. Furthermore, the effect of assay type on seroprevalence was investigated through meta-regression analyses.

Precision was classified as low or high based on the sample size, with studies having a sample size of less than 100 categorized as low precision, while those with 100 or more were considered high precision (Methods S1). For the ROB assessment, studies were classified as having low or high ROB based on the sampling method (probability-based or non-probability-based) and the response rate (80% or higher versus less than 80% or unclear). These assessments were subsequently used to provide summary statistics of the precision and ROB of the studies. These variables were also included in the meta-regression analyses to investigate their impact on observed seroprevalence, following an established methodology.^19^^,^^20^^,^^21^^,^^22^^,^^23^^,^^24^^,^^25^^,^^40^^,^^41^^,^^42^^,^^43^^,^^44^^,^^45^^,^^46^

Publication bias in meta-analyses was evaluated using Doi plots and LFK index whenever the number of pooled studies exceeded three.^56^ An asymmetrical Doi plot indicated potential publication bias; the spread of the outcome measures may not be due to chance alone.^56^ An LFK index value exceeding ±1 was considered indicative of the presence of publication bias.^56^

##### Meta-analyses

All meta-analyses were performed using R version 4.0.4,^57^ utilizing the "meta" package,^58^ as outlined in Methods S1. To address both sampling variation and heterogeneity in effect size, the DerSimonian-Laird random-effects model^59^ was employed, along with the Freeman-Tukey double arcsine transformation to stabilize the variance,^60^ after considering its validity for the study dataset.^61^ The meta-analyses were conducted to derive pooled mean estimates and 95% CIs for HSV-1 seroprevalence and the proportions of HSV-1 detection in both GUD and genital herpes. These pooled estimates were meant to provide an average summary of these measures across the included studies.

##### Meta-regressions

The study employed both univariable and multivariable random-effects meta-regression analyses to investigate the reasons for variation between studies and identify factors that may influence higher seroprevalence and HSV-1 genital herpes proportion. For informative meta-regression analyses, a minimum of 10 outcome measures were considered necessary. Log-transformed data for seroprevalence and HSV-1 genital herpes proportion were utilized, and both crude and adjusted relative risks (RRs), along with their corresponding 95% CIs, were presented.

Population-related and study methodology-related predictors were pre-selected based on their relevance and insights from prior research^19^^,^^20^^,^^21^^,^^22^^,^^23^^,^^24^^,^^25^ (Methods S1). In the univariable analysis, variables with a *p*-value≤0.10 were included in the subsequent multivariable analysis. Associations with a *p*-value≤0.05 in the multivariable analysis were deemed to be statistically significant.

The meta-regressions were conducted using the "metareg" package^62^ in Stata/SE version 17.^63^

### Quantification and Statistical analysis

Statistical analyses are described in the STAR methods section of the main text.

### Additional resources

The study did not generate or contribute to a new website/forum, nor is it part of a clinical trial.

## Supplemental information

Document S1. Figures S1–S4, Tables S2–S9, and Methods S1

Table S1. Studies reporting HSV-1 seroprevalence among different populations in the United States, related to Table 1
