# Supplementary material for: Epidemiology of herpes simplex virus type 1 in the United States: Systematic review, meta-analyses, and meta-regressions
Source: iScience. 2024 Aug 5;27(9):110652. doi: 10.1016/j.isci.2024.110652 (PMC11367537; doi:10.1016/j.isci.2024.110652)
Supplement: Document S1. Figures S1–S4, Tables S2–S9, and Methods S1 [file mmc1.pdf]

iScience, Volume *27*

Supplemental information

Epidemiology of herpes simplex virus type 1 in the United States: Systematic review, meta-analyses, and meta-regressions

Rwedah A. Ageeb, Manale Harfouche, Hiam Chemaitelly, and Laith J. Abu-Raddad

Supplemental Information

Table of Contents

Table S2. Summary of the precision assessment and risk of bias assessment for the studies reporting HSV-1 seroprevalence in the United States, related to STAR Methods. 3

Table S3. Assessment of publication bias in HSV-1 seroprevalence and HSV-1 detection in laboratory-confirmed genital herpes studies using Doi plots and the LFK index, related to Tables 1 and 3 and STAR Methods. 4

Figure S2. Forest plots of the pooled mean HSV-1 seroprevalence among healthy children and adult general populations in the United States, related to Table 1.[a] 6

Figure S3. Forest plots of the pooled mean HSV-1 seroprevalence among clinical populations in the United States, related to Table 1.[a] 7

Table S4. Univariable and multivariable meta-regression analyses for HSV-1 seroprevalence in the United States using year of data collection as a categorical variable, related to Table 2 8

Table S5. Univariable and multivariable meta-regression analyses for HSV-1 seroprevalence in the United States, incorporating year of publication as both a linear term and a categorical variable. Age group, instead of age bracket, was utilized as the age variable in the analyses, related to Table 2. 9

Table S6. Studies reporting proportion of HSV-1 detection in clinically diagnosed genital ulcer disease and proportion of HSV-1 detection in laboratory-confirmed genital herpes in the United States, related to Table 3. 10

Figure S4. Forest plots for the pooled mean proportion of HSV-1 detection in laboratory-confirmed genital herpes in the United States, related to Table 3.[a] 13

Table S7. Preferred Reporting Items for Systematic Reviews and Meta-analyses (PRISMA) checklist,[1] related to Figure 1 and STAR Methods. 14

Table S8. Data sources and search criteria for systematically reviewing HSV-1 epidemiology in the United States, related to STAR Methods. 17

Methods S1. Description of study methodology, related to STAR Methods. 18

Table S9. Range of quality components applicable to prevalence studies and their applicability to this systematic review study,[2] related to Tables 2 and 4 and STAR Methods. 19

Table S2. Summary of the precision assessment and risk of bias assessment for the studies reporting HSV-1 seroprevalence in the United States, related to STAR Methods.

Quality assessment	HSV-1 seroprevalence measures	
	Number of studies	%
**Precision of seroprevalence measures**[a]		
Low precision	26	13.7
High precision	164	86.3
**Risk of bias quality domain**[b]		
**Sampling methodology**		
Low risk of bias	74	38.9
High risk of bias	116	61.1
**Response rate**		
Low risk of bias	27	14.2
High risk of bias	45	23.7
Unclear risk of bias	118	62.1
**Summary of the risk of bias assessment**		
**Low risk of bias**		
In at least one quality domain	79	41.6
In both quality domains	22	11.6
**High risk of bias**		
In at least one quality domain	152	80.0
In both quality domains	9	4.7
**Unclear risk of bias**		
In at least one quality domain	118	62.1
In both quality domains	0	0.0
**Seroprevalence studies where risk of bias assessment was possible**	**190**	**100.0**

HSV-1: Herpes simplex virus type 1.
[a]Precision was assessed based on the overall sample size (not each stratum subsample size) of the study as reported in the publication.
[b]Risk of bias was assessed based on the overall sample size (not each stratum subsample size) of the study as reported in the publication.

Table S3. Assessment of publication bias in HSV-1 seroprevalence and HSV-1 detection in laboratory-confirmed genital herpes studies using Doi plots and the LFK index, related to Tables 1 and 3 and STAR Methods.

Population type	Total n	LFK index	Doi plot inspection	Interpretation
**HSV-1 seroprevalence studies**				
**Healthy general populations**				
Children	29	0.43	Slightly asymmetrical Doi plot	No publication bias
Adults	291	0.08	Symmetrical Doi plot	No publication bias
Age-mixed	11	-0.07	Slightly asymmetrical Doi plot	No publication bias
All healthy general populations	331	-0.45	Symmetrical Doi plot	No publication bias
**Clinal populations**				
Clinical adults	52	-0.43	Symmetrical Doi plot	No publication bias
Clinical age-mixed	8	1.11	Asymmetrical Doi plot	Indication of publication bias
All clinical populations	60	-0.6	Symmetrical Doi plot	No publication bias
**Other populations**				
HIV positive patients	14	-4.92	Asymmetrical Doi plot	Indication of publication bias
Men who have sex with men	8	-2.12	Slightly asymmetrical Doi plot	Indication of publication bias
Mixed population at risk	3	-2.99	Asymmetrical Doi plot	Indication of publication bias
Partners of genital herpes patients	4	-1.12	Symmetrical Doi plot	Indication of publication bias
Population exposed to sexual abuse	3	3.96	Asymmetrical Doi plot	Indication of publication bias
Women who have sex with women	4	2.34	Asymmetrical Doi plot	Indication of publication bias
**Specific sub-populations of epidemiological relevance**				
Healthy pregnant women	11	-4.43	Asymmetrical Doi plot	Indication of publication bias
HSV-2 positive patients	3	5.47	Asymmetrical Doi plot	Indication of publication bias
STD clinic attendees	22	-1.15	Slightly asymmetrical Doi plot	Indication of publication bias
**HSV-1 detection in laboratory-confirmed genital herpes studies**				
**Patients with laboratory-confirmed genital herpes**	51	-1.00	Symmetrical Doi plot	No publication bias

HIV: Human immunodeficiency virus; HSV-1: Herpes simplex virus type 1; HSV-2: Herpes simplex virus type 2; LFK: Luis Furuya-Kanamori; STD: Sexually transmitted disease.

Figure S1. Doi plots for assessment of publication bias, related to STAR Methods. A) Healthy children population. B) Healthy adult population. C) Healthy age-mixed population. D) All healthy general populations. E) Clinical adult population. F) Clinical age-mixed population. G) All clinical populations. H) HIV positive patients. I) Men who have sex with men. J) Mixed populations at risk. K) Partners of genital herpes patients. L) population exposed to sexual abuse. M) Women who have sex with women. N) Healthy pregnant women. O) HSV-2 positive patients. P) STD clinic attendees. Q) Patients with laboratory-confirmed genital herpes.

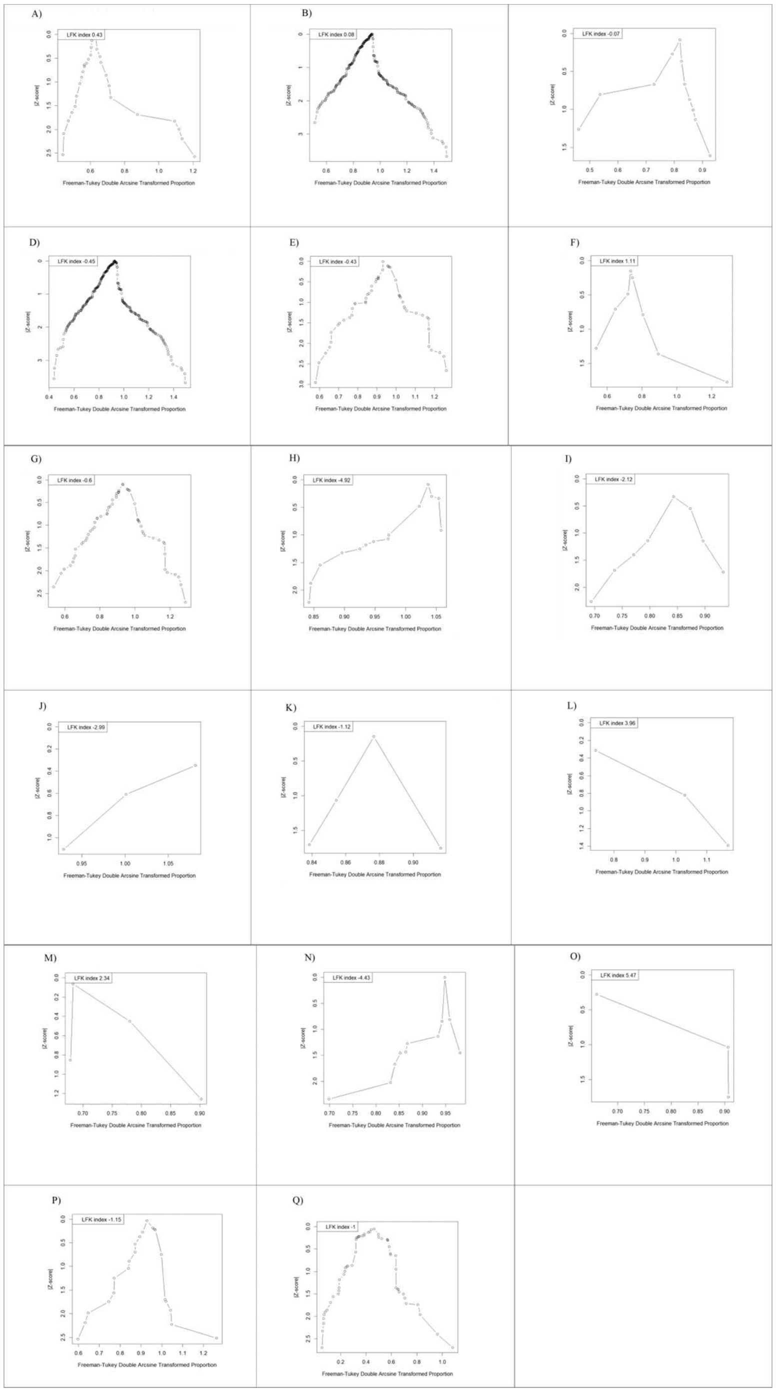

HIV: Human immunodeficiency virus; HSV-1: Herpes simplex virus type 1; HSV-2: Herpes simplex virus type 2; STD: Sexually transmitted disease.

Figure S2. Forest plots of the pooled mean HSV-1 seroprevalence among healthy children and adult general populations in the United States, related to Table 1.[a]

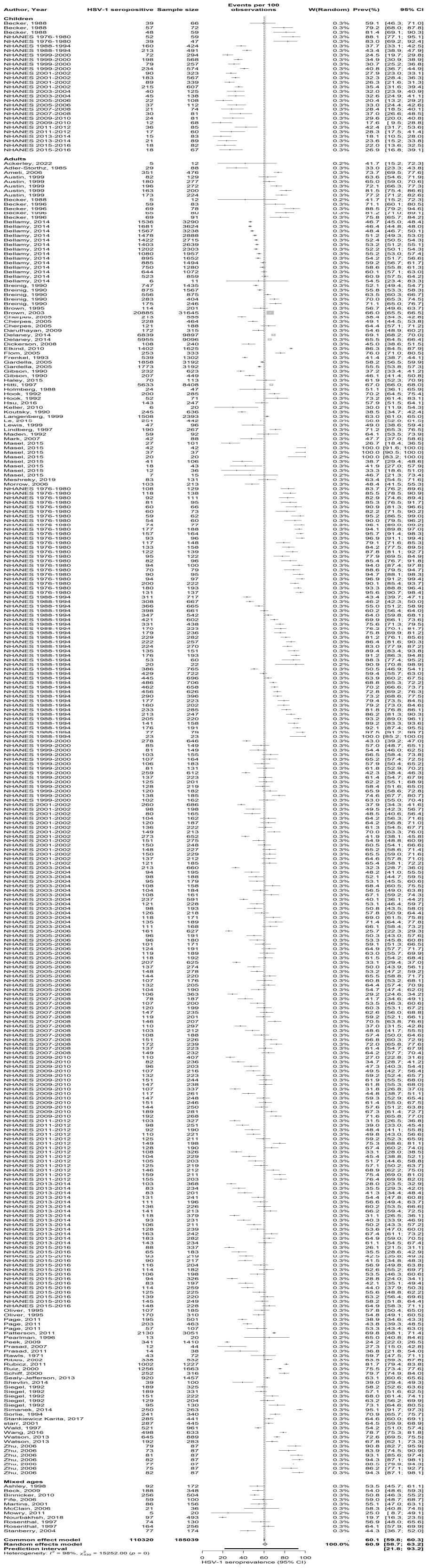

[a]Each line represents an HSV-1 seroprevalence measure in a specific stratum.

Figure S3. Forest plots of the pooled mean HSV-1 seroprevalence among clinical populations in the United States, related to Table 1.[a]

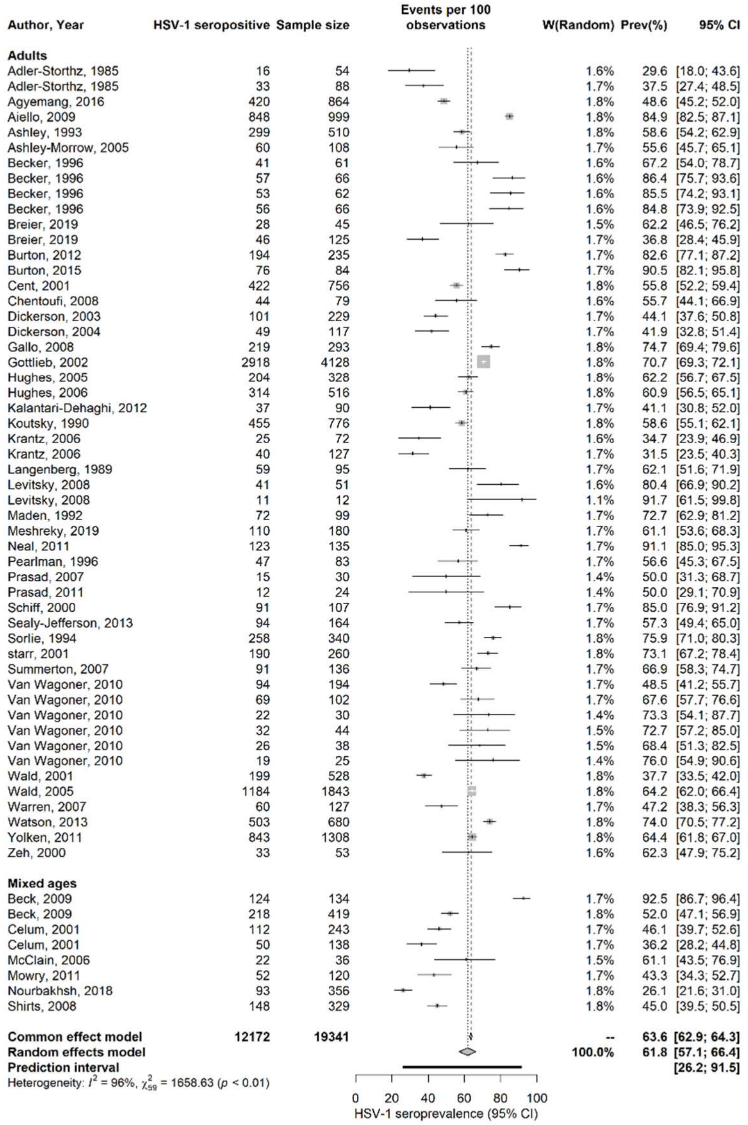

[a]Each line represents an HSV-1 seroprevalence measure in a specific stratum.

Table S4. Univariable and multivariable meta-regression analyses for HSV-1 seroprevalence in the United States using year of data collection as a categorical variable, related to Table 2.

			Outcome measures	Samples	Univariable analysis				Multivariable analysis			
									Model 1[a]		Model 2[b]	
			Total n	Total N	*RR* (95%CI)	p-value	LR test p-value	Adjusted R^2 (%)	*ARR* (95%CI)	p-value	*ARR* (95%CI)	p-value
Population Characteristics	**Age bracket**	Children	31	6,314	1.00	-	<0.001	13.35	-	-	1.00	-
		Adults	376	204,605	1.61 (1.43-1.81)	<0.001			-	-	1.39 (1.24-1.56)	<0.001
		Age-mixed	20	4,225	1.32 (1.10-1.58)	0.002			-	-	1.18 (0.99-1.40)	0.063
	**Age group**	<10	5	1,279	1.00	-	<0.001	43.44	1.00	-	-	-
		10-19	54	23,798	1.21 (0.96-1.54)	0.112			1.28 (1.02-1.59)	0.030	-	-
		20-29	75	37,807	1.73 (1.36-2.19)	<0.001			1.75 (1.40-2.19)	<0.001	-	-
		30-39	49	12,038	1.98 (1.56-2.52)	<0.001			2.11 (1.68-2.65)	<0.001	-	-
		40-49	45	9,985	2.12 (1.67-2.70)	<0.001			2.28 (1.81-2.88)	<0.001	-	-
		≥50	34	7,166	2.74 (2.15-3.49)	<0.001			2.50 (1.98-3.16)	<0.001	-	-
		Mixed	165	123,071	1.89 (1.50-2.39)	<0.001			1.80 (1.43-2.25)	<0.001	-	-
	**Sex**	Female	188	143,121	1.00	-	0.310[c]	0.06	1.00	-	1.00	-
		Male	145	33,087	0.95 (0.89-1.02)	0.183			0.96 (0.92-1.01)	0.153	0.97 (0.91-1.03)	0.296
		Mixed	94	38,936	1.01 (0.93-1.09)	0.843			1.01 (0.94-1.08)	0.820	1.05 (0.97-1.13)	0.229
	**Population type**	Healthy	331	185,039	1.00	-	0.568	0.29	-	-	-	-
		Clinical	60	19,341	1.03 (0.94-1.13)	0.508			-	-	-	-
		Other	36	10,764	1.05 (0.94-1.18)	0.366			-	-	-	-
Study methodology characteristics	**Assay type**	Western blot	117	136,170	1.00	-	0.265	0.14	-	-	-	-
		ELISA	303	77,725	0.95 (0.89-1.02)	0.149			-	-	-	-
		Others	7	1,249	0.87 (0.67-1.13)	0.305			-	-	-	-
	**Sample size[d]**	<100	27	1,385	1.00	-	0.075	0.26	1.00	-	1.00	-
		≥100	400	213,759	1.13 (0.99-1.30)	0.075			1.15 (1.03-1.29)	0.011	1.18 (1.04-1.33)	0.010
	**Sampling method**	Probability-based	253	69,851	1.00	-	0.165	0.22	-	-	-	-
		Non-probability-based	174	145,293	1.05 (0.98-1.12)	0.152			-	-	-	-
	**Response rate**	≥80	58	25,507	1.00	-	<0.001	11.90	1.00		1.00	-
		<80	193	58,015	1.39 (1.27-1.53)	<0.001			1.01 (0.93-1.10)	0.861	1.25 (1.14-1.36)	<0.001
		Unclear	176	131,622	1.33 (1.21-1.46)	<0.001			1.10 (1.00-1.20)	0.039	1.21 (1.11-1.33)	<0.001
Temporal variable	**Year of data collection category**	<1995	144	109,388	1.00	-	<0.001	17.43	1.00	-	1.00	-
		1995-2004	140	64,309	0.80 (0.75-0.86)	<0.001			0.87 (0.82-0.92)	<0.001	0.83 (0.78-0.88)	<0.001
		≥2005	143	41,447	0.73 (0.68-0.78)	<0.001			0.78 (0.74-0.83)	<0.001	0.75 (0.70-0.79)	<0.001

ARR: Adjusted risk ratio; CI: Confidence interval; ELISA: Enzyme-linked immunosorbent type-specific assay; HSV-1: Herpes simplex virus type 1; *RR*: Risk ratio.
[a]Variance explained by the final multivariable model 1 (adjusted R2) = 53.06%.
[b]Variance explained by the final multivariable model 2 (adjusted R2) = 34.00%.
[c]Although sex variable did not have a statistically significant association with the outcome in the univariable analysis (p-value>0.1), it was included in the multivariable analysis because of epidemiological relevance.
[d]Sample size denotes the sample size of the study population found in the original publication.

Table S5. Univariable and multivariable meta-regression analyses for HSV-1 seroprevalence in the United States, incorporating year of publication as both a linear term and a categorical variable. Age group, instead of age bracket, was utilized as the age variable in the analyses, related to Table 2.

			Outcome measures	Samples	Univariable analysis				Multivariable analysis			
									Model 1[a]		Model 2[b]	
			Total n	Total N	*RR* (95%CI)	p-value	LR test p-value	Adjusted R^2 (%)	*ARR* (95%CI)	p-value	*ARR* (95%CI)	p-value
Population Characteristics	Age bracket	Children	31	6,314	1.00	-	<0.001	13.35	-	-	-	-
		Adults	376	204,605	1.61 (1.43-1.81)	<0.001			-	-	-	-
		Age-mixed	20	4,225	1.32 (1.10-1.58)	0.002			-	-	-	-
	Age group	<10	5	1,279	1.00	-	<0.001	43.44	1.00	-	1.00	-
		10-19	54	23,798	1.21 (0.96-1.54)	0.112			1.26 (1.01-1.57)	0.045	1.30 (1.04-1.61)	0.019
		20-29	75	37,807	1.73 (1.36-2.19)	<0.001			1.74 (1.38-2.18)	<0.001	1.81 (1.45-2.25)	<0.001
		30-39	49	12,038	1.98 (1.56-2.52)	<0.001			2.07 (1.64-2.60)	<0.001	2.14 (1.71-2.68)	<0.001
		40-49	45	9,985	2.12 (1.67-2.70)	<0.001			2.24 (1.77-2.83)	<0.001	2.31 (1.83-2.90)	<0.001
		≥50	34	7,166	2.74 (2.15-3.49)	<0.001			2.52 (1.99-3.19)	<0.001	2.58 (2.05-3.24)	<0.001
		Mixed	165	123,071	1.89 (1.50-2.39)	<0.001			1.79 (1.43-2.24)	<0.001	1.86 (1.49-2.31)	<0.001
	Sex	Female	188	143,121	1.00	-	0.310[c]	0.06	1.00	-	1.00	-
		Male	145	33,087	0.95 (0.89-1.02)	0.183			0.95 (0.90-1.00)	0.060	0.95 (0.90-0.99)	0.027
		Mixed	94	38,936	1.01 (0.93-1.09)	0.843			1.00 (0.93-1.07)	0.928	0.99 (0.93-1.06)	0.877
	Population type	Healthy	331	185,039	1.00	-	0.568	0.29	-	-	-	-
		Clinical	60	19,341	1.03 (0.94-1.13)	0.508			-	-	-	-
		Other	36	10,764	1.05 (0.94-1.18)	0.366			-	-	-	-
Study methodology characteristics	Assay type	Western blot	117	136,170	1.00	-	0.265	0.14	-	-	-	-
		ELISA	303	77,725	0.95 (0.89-1.02)	0.149			-	-	-	-
		Others	7	1,249	0.87 (0.67-1.13)	0.305			-	-	-	-
	Sample size[d]	<100	27	1,385	1.00	-	0.075	0.26	1.00	-	1.00	-
		≥100	400	213,759	1.13 (0.99-1.30)	0.075			1.21 (1.08-1.35)	0.001	1.23 (1.11-1.38)	<0.001
	Sampling method	Probability-based	253	69,851	1.00	-	0.165	0.22	-	-	-	-
		Non-probability-based	174	145,293	1.05 (0.98-1.12)	0.152			-	-	-	-
	Response rate	≥80	58	25,507	1.00	-	<0.001	11.90	1.00	-	1.00	-
		<80	193	58,015	1.39 (1.27-1.53)	<0.001			1.00 (0.92-1.10)	0.917	1.01 (0.93-1.10)	0.802
		Unclear	176	131,622	1.33 (1.21-1.46)	<0.001			1.13 (1.03-1.24)	0.009	1.16 (1.06-1.27)	0.001
Temporal variable	Year of publication category	<2000	134	46,690	1.00	-	<0.001	14.85	1.00	-	-	-
		2000-2009	155	85,186	0.79 (0.73-0.85)	<0.001			0.86 (0.81-0.91)	<0.001	-	-
		≥2010	138	83,268	0.75 (0.70-0.81)	<0.001			0.79 (0.74-0.84)	<0.001	-	-
	Year of publication as a linear term		427	215,144	0.99 (0.99-0.99)	<0.001	<0.001	15.14	-	-	0.99 (0.99-0.99)	<0.001

ARR: Adjusted risk ratio; CI: Confidence interval; ELISA: Enzyme-linked immunosorbent type-specific assay; HSV-1: Herpes simplex virus type 1; *RR*: Risk ratio.

[a]Variance explained by the final multivariable model 1 (adjusted R^2) = 52.12%.

[b]Variance explained by the final multivariable model 2 (adjusted R^2) = 55.35%.

[c]Although sex variable did not have a statistically significant association with the outcome in the univariable analysis (p-value>0.1), it was included in the multivariable analysis because of epidemiological relevance.

[d]Sample size denotes the sample size of the study population found in the original publication.

Table S6. Studies reporting proportion of HSV-1 detection in clinically diagnosed genital ulcer disease and proportion of HSV-1 detection in laboratory-confirmed genital herpes in the United States, related to Table 3.

Author, Year[a]	Year(s) of data collection	City, province	Study site	Study design[b]	Sampling methodology	HSV-1 biological assay	Population[c]	Population age		Sample size	Proportion of HSV-1 detection (%)
								Median/ Mean (SD)	Range		
**Patients with clinically diagnosed genital ulcer disease**											
Van Der Pol, 2012[1]	-	-	Outpatient clinic	CS	Conv	PCR	Patients with genital lesions	-	≥18	506	14.2
Binnicker, 2017[2]	2005	-	Outpatient clinic	CS	Conv	PCR	Patients with genital lesions	-	-	193	22.8
**Patients with laboratory-confirmed genital herpes**											
Chang, 1974[3]	1971-72	Boston, Massachusetts	Outpatient clinic	CS	Conv	Culture	Patients with first episode genital herpes	-	16-45	21	52.4
Kalinyak, 1977[4]	-	-	Outpatient clinic	Cohort	Conv	Culture	Women with herpetic lesions	-	18-23	30	37.0
Corey, 1978[5]	1976-77	-	Outpatient clinic	CS	Conv	Culture	Study participants with culture-proved primary genital herpes	25.0	≥14	71	2.8
Vontver, 1979[6]	1975-76	Seattle, Washington	Outpatient clinic	Cohort	Conv	Culture	Study participants with genital herpes	-	18-41	69	10.1
Boehm, 1981[7]	1976-80	Nashville, Tennessee	Hospital	Cohort	Conv	Culture	Pregnant women with suspected HSV infection	-	≥15	22	4.6
Reichman, 1982[8]	-	California	Hospital	RCT	Conv	Culture	Patients with recurrent genital herpes		18-50	107	0.0
Corey, 1982[9]	-	Washington; Atlanta, Georgia	Outpatient clinic	RCT	Conv	Culture	Patients with genital herpes	27.0	≥14	77	7.8
Scher, 1982[10]	1979-80	New York city, New York	Outpatient clinic	Cohort	Conv	Culture	Obstetric patients with symptomatic HSV infection	27.0	19-40	10	40.0
Bryson, 1983[11]	1981	Los Angeles, California	Outpatient clinic	RCT	Conv	Culture	Study participants with first episode genital herpes	25.0	≥18	48	10.4
Bryson, 1983[12]	1982-85	Los Angeles, California	Outpatient clinic	Cohort	Conv	Culture	Study male participants with clinical genital herpes	31.0	≥14	33	9.1
Bryson, 1983[12]	1982-85	Los Angeles, California	Outpatient clinic	Cohort	Conv	Culture	Study Female participants with clinical genital herpes	31.0	≥14	24	12.5
Corey, 1983[13]	-	Washington	Outpatient clinic	RCT	Conv	Culture	Patients with primary genital herpes	26.0	≥14	29	10.3
McLaren, 1983[14]	1979-80	Washington	Outpatient clinic	RCT	Conv	Culture	Study patients with first-episode or recurrent genital HSV infections	-	-	97	5.2
Mertz, 1985[15]	1979-81	Seattle, Washington	Outpatient clinic	CS	Conv	Culture	Partners of genital herpes patients with suspected genital lesions	28.2	≥15	66	7.6
Lafferty, 1987[16]	1975-76	Seattle, Washington	Outpatient clinic	CS	Conv	Culture	Study participants with primary genital herpes	25.6	≥14	39	30.8
Levin, 1989[17]	1983-85	Denver, Colorado; Los Angeles, California; Seattle, Washington	Outpatient clinic	RCT	Conv	Culture	Patients with primary and non-primary genital herpes	-	≥18	105	17.1
Ashley, 1991[18]	-	-	Outpatient clinic	CS	Conv	Culture	Study participants with primary genital herpes	-	-	28	42.9
Ashley, 1991[18]	-	-	Outpatient clinic	CS	Conv	Culture	Study participants with non-primary genital herpes	-	-	12	0.0
Ashley, 1991[18]	-	-	Outpatient clinic	CS	Conv	Culture	Study participants with recurrent genital herpes	-	-	50	10.0
Koelle, 1992[19]	1978-88	Washington	Outpatient clinic	Cohort	Conv	Culture	Women with primary genital herpes	22.0	20-27	306	14.1
Lebwohl, 1992[20]	-	-	Outpatient clinic	CS	Conv	Culture	Patients with recurrent genital lesions	30.5	17-67	307	1.3

Author, Year[a]	Year(s) of data collection	City, province	Study site	Study design[b]	Sampling methodology	HSV-1 biological assay	Population[c]	Population age		Sample size	Proportion of HSV-1 detection (%)
								Median/ Mean (SD)	Range		
Mertz, 1992[21]	-	Washington; New Mexico	Outpatient clinic	Cohort	Conv	Culture	Susceptible partners of patients with genital herpes	-	≥18	12	8.3
Benedetti, 1994[22]	1974-88	Seattle, Washington	Community	Cohort	Conv	Culture	Patients with primary genital HSV infection	24.0	-	380	18.2
Benedetti, 1994[22]	1974-88	Seattle, Washington	Community	Cohort	Conv	Culture	Patients with recurrent HSV infection	24.0	-	56	0.0
Skinner, 1997[23]	1987-88	Chicago, Illinois; Houston, Texas; Los Angeles, California	Outpatient clinic	RCT	Conv	Culture	Study participants with history of recurrent HSV disease	32.0	≥18	71	0.0
Benedetti, 1999[24]	1974-91	Washington	Outpatient clinic	Cohort	Conv	Culture	Patients with primary genital herpes	-	16-51	265	22.6
Lafferty, 2000[25]	1993-97	Seattle, Washington	Outpatient clinic	CS	Conv	Culture	Patients with genital herpes	-	≥14	1,145	17.1
Reyes, 2002[26]	1996-98	Atlanta, Georgia; Birmingham, Alabama; Chicago, Illinois; Columbus, Ohio; Denver, Colorado; Houston, Texas; Indianapolis, Indiana; Nassau Bay, Texas; New Orleans, Louisiana; New York City, New York; San Francisco, California; Seattle, Washington	Outpatient clinic	CS	Conv	Mab	Study participants suspected of having active genital herpes	31.0	18-93	2,088	9.8
Brown, 2003[27]	1982-99	Seattle, Washington; Tacoma, Washington	Hospital	CS	Conv	PCR	Pregnant women with genital lesions	-	≥15	56	5.4
Cannon, 2003[28]	1996-98	Atlanta, Georgia; Birmingham, Alabama; Chicago, Illinois; Columbus, Ohio; Denver, Colorado; Houston, Texas; Indianapolis, Indiana; Nassau Bay, Texas; New Orleans, Louisiana; New York City, New York; San Francisco, California; Seattle, Washington	Outpatient clinic	CS	Conv	Culture	Study participants with recurrent genital herpes	-	≥18	940	4.2
Roberts, 2003[29]	1993-01	Midwest region in the United States	Outpatient clinic	CS	Conv	Culture	Patients with primary genital herpes	-	≥16	499	48.9
Hughes, 2006[30]	-	Birmingham, Alabama	Outpatient clinic	CS	Conv	PCR	Men with genital herpes	25.0	18-68	39	10.3
Handsfield, 2007[31]	2001-02	Seattle, Washington; Portland, Oregon	Outpatient clinic	RCT	Conv	PCR	Patients with first episode genital herpes	28.7	≥18	45	28.9
Meng, 2008[32]	2002-03	-	Outpatient clinic	RCT	Conv	PCR	Patients with history of genital herpes	39.0 (10.0)	18-65	82	0.0
Wald, 2008[33]	1996-02	Seattle, Washington	Outpatient clinic	CS	Conv	PCR	Patients with signs and symptoms of genital herpes	-	≥14	236	33.0

Author, Year[a]	Year(s) of data collection	City, province	Study site	Study design[b]	Sampling methodology	HSV-1 biological assay	Population[c]	Population age		Sample size	Proportion of HSV-1 detection (%)
								Median/ Mean (SD)	Range		
Horowitz, 2010[34]	2004-07	Northeastern region in the United States	Outpatient clinic	CS	Conv	PCR	Patients with herpetic lesions	-	≥18	125	71.2
Van Wagoner, 2010[35]	-	Birmingham, Alabama	Outpatient clinic	CC	Conv	PCR	Circumcised black heterosexual men attending STD clinic	-	≥18	14	14.3
Katsumata, 2012[36]	2007-08	-	Outpatient clinic	RCT	Conv	Culture	Patients with genital herpes	-	-	156	3.2
Tyring, 2012[37]	2007-08	-	Outpatient clinic	RCT	Conv	Culture	Participants with recurrent genital herpes	-	≥18	167	3.0
Van Der Pol, 2012[1]	-	-	Outpatient clinic	CS	Conv	PCR	Patients with genital lesions	-	≥18	315	22.9
Young, 2015[38]	-	-	Outpatient clinic	RCT	Conv	PCR	Patients with genital herpes	-	-	243	34.6
Faron, 2016[39]	-	-	Outpatient clinic	CS	Conv	PCR	Patients with genital herpes	-	-	257	42.0
Dabestani, 2019[40]	1993-14	Seattle, Washington; King County, Washington	Outpatient clinic	CS	Conv	Mab	Patients with first episode genital herpes	-	≥18	3,065	33.3

CC: Case control; Conv: Convenience; CS: Cross sectional; HSV: Herpes simplex virus; HSV-1: Herpes simplex virus type 1; Mab: Monoclonal antibodies; PCR: Polymerase chain reaction; RCT: Randomized controlled trial; STD: Sexually transmitted disease.

[a]Studies are sorted by year of publication, in chronological order from earliest to latest.

[b]The reported study design is the original study design (cross-sectional, cohort, or randomized controlled trial). The included seroprevalence measures are those for the baseline measures at the beginning of the study.

[c]The included measures for the proportion of HSV-1 detection are those for the baseline measures at the beginning of the study population from which the study sample was derived and for which genital ulcer disease and genital herpes were assessed.

Figure S4. Forest plots for the pooled mean proportion of HSV-1 detection in laboratory-confirmed genital herpes in the United States, related to Table 3.[a]

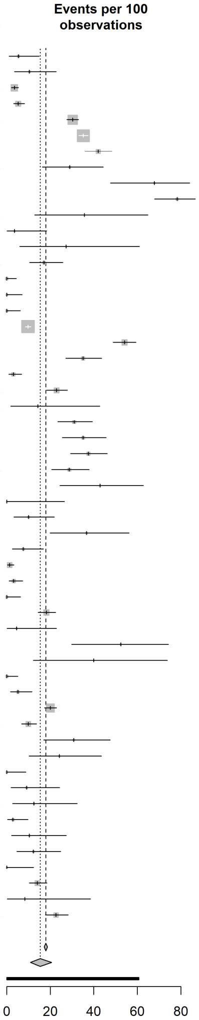

Author, Year	HSV-1 positive	Sample size	W(Random)	Prev(%)	95% CI
Brown, 2003	3	56	1.8%	5.4	[1.1; 14.9]
Bryson, 1983	5	48	1.8%	10.4	[3.5; 22.7]
Cannon, 2003	20	575	2.0%	3.5	[2.1; 5.3]
Cannon, 2003	19	360	2.0%	5.3	[3.2; 8.1]
Dabestani, 2019	349	1154	2.0%	30.2	[27.6; 33.0]
Dabestani, 2019	673	1911	2.0%	35.2	[33.1; 37.4]
Faron, 2016	108	257	2.0%	42.0	[35.9; 48.3]
Handsfield, 2007	13	45	1.8%	28.9	[16.4; 44.3]
Horowitz, 2010	19	28	1.7%	67.9	[47.6; 84.1]
Horowitz, 2010	65	83	1.9%	78.3	[67.9; 86.6]
Horowitz, 2010	5	14	1.5%	35.7	[12.8; 64.9]
Hughes, 2006	1	28	1.7%	3.6	[0.1; 18.3]
Hughes, 2006	3	11	1.4%	27.3	[6.0; 61.0]
Levin, 1989	18	105	1.9%	17.1	[10.5; 25.7]
Meng, 2008	0	82	1.9%	0.0	[0.0; 4.4]
Reichman, 1982	0	50	1.8%	0.0	[0.0; 7.1]
Reichman, 1982	0	57	1.8%	0.0	[0.0; 6.3]
Reyes, 2002	204	2088	2.0%	9.8	[8.5; 11.1]
Roberts, 2003	196	362	2.0%	54.1	[48.9; 59.4]
Roberts, 2003	48	137	1.9%	35.0	[27.1; 43.6]
Tyring, 2012	5	167	1.9%	3.0	[1.0; 6.8]
Van Der Pol, 2012	72	315	2.0%	22.9	[18.3; 27.9]
Van Wagoner, 2010	2	14	1.5%	14.3	[1.8; 42.8]
Wald, 2008	44	142	1.9%	31.0	[23.5; 39.3]
Wald, 2008	33	94	1.9%	35.1	[25.5; 45.6]
Young, 2015	51	136	1.9%	37.5	[29.4; 46.2]
Young, 2015	33	115	1.9%	28.7	[20.6; 37.9]
Ashley, 1991	12	28	1.7%	42.9	[24.5; 62.8]
Ashley, 1991	0	12	1.5%	0.0	[0.0; 26.5]
Ashley, 1991	5	50	1.8%	10.0	[3.3; 21.8]
Kalinyak, 1977	11	30	1.7%	36.7	[19.9; 56.1]
Mertz, 1985	5	66	1.9%	7.6	[2.5; 16.8]
Lebwohl, 1992	4	307	2.0%	1.3	[0.4; 3.3]
Katsumata, 2012	5	156	1.9%	3.2	[1.0; 7.3]
Benedetti, 1994	0	56	1.8%	0.0	[0.0; 6.4]
Benedetti, 1994	69	380	2.0%	18.2	[14.4; 22.4]
Boehm, 1981	1	22	1.7%	4.5	[0.1; 22.8]
Chang, 1974	11	21	1.6%	52.4	[29.8; 74.3]
Scher, 1982	4	10	1.4%	40.0	[12.2; 73.8]
Skinner, 1997	0	71	1.9%	0.0	[0.0; 5.1]
McLaren, 1983	5	97	1.9%	5.2	[1.7; 11.6]
Lafferty, 2000	164	821	2.0%	20.0	[17.3; 22.9]
Lafferty, 2000	32	324	2.0%	9.9	[6.9; 13.7]
Lafferty, 1987	12	39	1.8%	30.8	[17.0; 47.6]
Vontver, 1979	7	29	1.7%	24.1	[10.3; 43.5]
Vontver, 1979	0	40	1.8%	0.0	[0.0; 8.8]
Bryson, 1983	3	33	1.8%	9.1	[1.9; 24.3]
Bryson, 1983	3	24	1.7%	12.5	[2.7; 32.4]
Corey, 1978	2	71	1.9%	2.8	[0.3; 9.8]
Corey, 1983	3	29	1.7%	10.3	[2.2; 27.4]
Corey, 1982	6	49	1.8%	12.2	[4.6; 24.8]
Corey, 1982	0	28	1.7%	0.0	[0.0; 12.3]
Koelle, 1992	43	306	2.0%	14.1	[10.4; 18.5]
Mertz, 1992	1	12	1.5%	8.3	[0.2; 38.5]
Benedetti, 1999	60	265	2.0%	22.6	[17.7; 28.2]
**Common effect model**	**2457**	**11810**	**--**	**17.9**	**[17.2; 18.6]**
**Random effects model**			**100.0%**	**15.4**	**[10.8; 20.6]**
**Prediction interval**					**[0.0; 60.7]**

Heterogeneity: $I^2 = 97\%$, $\chi^2_{54} = 1820.75$ ($p = 0$)

[a]Each line represents a proportion measure of HSV-1 detection in genital herpes in a specific stratum.

Table S7. Preferred Reporting Items for Systematic Reviews and Meta-analyses (PRISMA) checklist,[41] related to Figure 1 and STAR Methods.

Section and Topic	Item #	Checklist item	Location where item is reported
**TITLE**			
Title	1	Identify the report as a systematic review.	p. 1
**ABSTRACT**			
Abstract	2	See the PRISMA 2020 for Abstracts checklist.	p. 2
**INTRODUCTION**			
Rationale	3	Describe the rationale for the review in the context of existing knowledge.	p. 3-4
Objectives	4	Provide an explicit statement of the objective(s) or question(s) the review addresses.	p. 3-4
**METHODS**			
Eligibility criteria	5	Specify the inclusion and exclusion criteria for the review and how studies were grouped for the syntheses.	p. 23; Methods S1
Information sources	6	Specify all databases, registers, websites, organisations, reference lists and other sources searched or consulted to identify studies. Specify the date when each source was last searched or consulted.	p. 22 Methods S1
Search strategy	7	Present the full search strategies for all databases, registers and websites, including any filters and limits used.	Table S8
Selection process	8	Specify the methods used to decide whether a study met the inclusion criteria of the review, including how many reviewers screened each record and each report retrieved, whether they worked independently, and if applicable, details of automation tools used in the process.	p. 23; Methods S1
Data collection process	9	Specify the methods used to collect data from reports, including how many reviewers collected data from each report, whether they worked independently, any processes for obtaining or confirming data from study investigators, and if applicable, details of automation tools used in the process.	p. 24; Methods S1
Data items	10a	List and define all outcomes for which data were sought. Specify whether all results that were compatible with each outcome domain in each study were sought (e.g. for all measures, time points, analyses), and if not, the methods used to decide which results to collect.	Methods S1
	10b	List and define all other variables for which data were sought (e.g. participant and intervention characteristics, funding sources). Describe any assumptions made about any missing or unclear information.	Methods S1
Study risk of bias assessment	11	Specify the methods used to assess risk of bias in the included studies, including details of the tool(s) used, how many reviewers assessed each study and whether they worked independently,	p. 24-26; Methods S1

Section and Topic	Item #	Checklist item	Location where item is reported
		and if applicable, details of automation tools used in the process.	
Effect measures	12	Specify for each outcome the effect measure(s) (e.g. risk ratio, mean difference) used in the synthesis or presentation of results.	Methods S1
Synthesis methods	13a	Describe the processes used to decide which studies were eligible for each synthesis (e.g. tabulating the study intervention characteristics and comparing against the planned groups for each synthesis (item #5)).	p. 23-24;<br>Methods S1
	13b	Describe any methods required to prepare the data for presentation or synthesis, such as handling of missing summary statistics, or data conversions.	Methods S1
	13c	Describe any methods used to tabulate or visually display results of individual studies and syntheses.	Methods S1
	13d	Describe any methods used to synthesize results and provide a rationale for the choice(s). If meta-analysis was performed, describe the model(s), method(s) to identify the presence and extent of statistical heterogeneity, and software package(s) used.	p. 26-27; Methods S1
	13e	Describe any methods used to explore possible causes of heterogeneity among study results (e.g. subgroup analysis, meta-regression).	p. 27; Methods S1
	13f	Describe any sensitivity analyses conducted to assess robustness of the synthesized results.	p. 27
Reporting bias assessment	14	Describe any methods used to assess risk of bias due to missing results in a synthesis (arising from reporting biases).	NA
Certainty assessment	15	Describe any methods used to assess certainty (or confidence) in the body of evidence for an outcome.	NA
**RESULTS**			
Study selection	16a	Describe the results of the search and selection process, from the number of records identified in the search to the number of studies included in the review, ideally using a flow diagram.	p. 4; Figure 1
	16b	Cite studies that might appear to meet the inclusion criteria, but which were excluded, and explain why they were excluded.	Figure 1
Study characteristics	17	Cite each included study and present its characteristics.	Tables S1 and S6
Risk of bias in studies	18	Present assessments of risk of bias for each included study.	p. 5-6; Tables S2, S3 and S9; Figure S1
Results of individual studies	19	For all outcomes, present, for each study: (a) summary statistics for each group (where appropriate) and (b) an effect estimate and its precision (e.g. confidence/credible interval), ideally using structured tables or plots.	p. 6-8; Tables 1 and 3; Figures S2, S3 and S4; Tables S1 and S6
Results of syntheses	20a	For each synthesis, briefly summarise the characteristics and risk of bias among contributing	Figure S1; Tables S1,

Section and Topic	Item #	Checklist item	Location where item is reported
		studies.	S6, S2, S3 and S9
	20b	Present results of all statistical syntheses conducted. If meta-analysis was done, present for each the summary estimate and its precision (e.g. confidence/credible interval) and measures of statistical heterogeneity. If comparing groups, describe the direction of the effect.	p. 6-8; Tables 1 and 3
	20c	Present results of all investigations of possible causes of heterogeneity among study results.	p. 6-8; Tables 2 and 4; Table S4, S5
	20d	Present results of all sensitivity analyses conducted to assess the robustness of the synthesized results.	Tables S4 and S5
Reporting biases	21	Present assessments of risk of bias due to missing results (arising from reporting biases) for each synthesis assessed.	NA
Certainty of evidence	22	Present assessments of certainty (or confidence) in the body of evidence for each outcome assessed.	NA
**DISCUSSION**			
Discussion	23a	Provide a general interpretation of the results in the context of other evidence.	p. 8-11
	23b	Discuss any limitations of the evidence included in the review.	p. 11-12
	23c	Discuss any limitations of the review processes used.	p. 11-12
	23d	Discuss implications of the results for practice, policy, and future research.	p. 11
**OTHER INFORMATION**			
Registration and protocol	24a	Provide registration information for the review, including register name and registration number, or state that the review was not registered.	NA
	24b	Indicate where the review protocol can be accessed, or state that a protocol was not prepared.	NA
	24c	Describe and explain any amendments to information provided at registration or in the protocol.	NA
Support	25	Describe sources of financial or non-financial support for the review, and the role of the funders or sponsors in the review.	p. 13
Competing interests	26	Declare any competing interests of review authors.	p. 13
Availability of data, code and other materials	27	Report which of the following are publicly available and where they can be found: template data collection forms; data extracted from included studies; data used for all analyses; analytic code; any other materials used in the review.	p. 21 and Key resources table (KRT)

NA, not applicable; p, page(s).

Table S8. Data sources and search criteria for systematically reviewing HSV-1 epidemiology in the United States, related to STAR Methods.

**PubMed (last searched April 10, 2023):**
(“Simplexvirus”[MeSH] OR “Herpes Simplex”[MeSH]) OR (HSV type-1[Text] OR HSV type 1[Text] OR HSV1[Text] OR HSV-1[Text] OR HSV 1[Text] OR Human herpes virus[Text] OR Herpes simplex virus type 1[Text] OR Herpes simplex virus type-1[Text] OR herpes simplex virus 1[Text] OR herpes simplex virus-1[Text] OR herpes simplex type 1[Text] OR herpes simplex type-1[Text] OR herpes simplex 1[Text] OR herpes simplex-1[Text] OR Herpesvirus type 1[Text] OR Herpesvirus type-1[Text] OR Herpesvirus 1[Text] OR Herpesvirus-1[Text] OR Herpes virus type 1[Text] OR Herpes virus type-1[Text] OR Herpes virus 1[Text] OR Herpes virus-1[Text] OR genital herpes[Text] OR Herpes Genitalis[Text] OR Stomatitis Herpetic[Text] OR Herpes Labialis[Text]) AND (“United States”[Mesh]) OR (United States[Text] OR United States of America[Text] OR America[Text] OR USA[Text] OR US[Text] OR U.S.A[Text] OR U.S.[Text] OR The States[Text] OR Appalachian Region[Text] OR Alabama[Text] OR Kentucky[Text] OR Maryland[Text] OR New York[Text] OR Ohio [Text] OR Pennsylvania[Text] OR Tennessee[Text] OR Virginia[Text] OR West Virginia[Text] OR Great Lakes Region[Text] OR Illinois[Text] OR Indiana[Text] OR Michigan[Text] OR Minnesota[Text] OR Wisconsin[Text] OR Mid-Atlantic Region[Text] OR Delaware[Text] OR District of Columbia[Text] OR New Jersey[Text] OR Midwestern United States[Text] OR Iowa[Text] OR Kansas[Text] OR Missouri[Text] OR Nebraska[Text] OR North Dakota[Text] Oklahoma[Text] OR South Dakota[Text] OR New England[Text] OR Connecticut[Text] OR Maine[Text] OR Massachusetts[Text] OR New Hampshire[Text] OR Rhode Island[Text] OR Vermont[Text] OR Northwestern United States[Text] OR Idaho[Text] OR Montana[Text] OR Oregon[Text] OR Washington[Text] OR Wyoming[Text] OR Pacific States[Text] OR Alaska[Text] OR California[Text] OR Hawaii [Text] OR Southeastern United States[Text] OR Arkansas[Text] OR Florida[Text] OR Georgia[Text] OR Louisiana[Text] OR Mississippi[Text] OR North Carolina[Text] OR South Carolina[Text] OR Southwestern United States[Text] OR Arizona[Text] OR Colorado[Text] OR Nevada[Text] OR New Mexico[Text] OR Texas[Text] OR Utah[Text] OR American*[Text])
**Embase (last searched April 10, 2023):**
(exp Herpes simplex virus/ or exp herpes simplex/ or (Herpes simplex or Herpes simplex virus or HSV type-1 or HSV type 1 or HSV1 or HSV-1 or HSV 1 or human herpes virus or Herpes simplex virus type 1 or Herpes simplex virus type-1 or herpes simplex virus 1 or herpes simplex virus-1 or herpes simplex type 1 or herpes simplex type-1 or herpes simplex 1 or herpes simplex-1 or Herpesvirus type 1 or Herpesvirus type-1 or Herpesvirus 1 or Herpesvirus-1 or Herpes virus type 1 or Herpes virus type-1 or Herpes virus 1 or Herpes virus-1 or genital herpes or Herpes Genitalis or herpes labialis or herpetic stomatitis).mp.) AND (exp United States/ or ("United States" or "United States of America" or America or USA or US or "The States" or "Appalachian region" or Kentucky or Maryland or "New York" or Ohio or Pennsylvania or Tennessee or Virginia or "West Virginia" or "Great Lakes Region" or Illinois or Indiana or Michigan or Minnesota or Wisconsin or "Mid-Atlantic Region" or Delaware or "District of Columbia" or "New Jersey" or "Midwestern United States" or Iowa or Kansas or Missouri or Nebraska or "North Dakota" or Oklahoma or "South Dakota" or "New England" or Connecticut or Maine or Massachusetts or "New Hampshire" or "Rhode Island" or Vermont or "Northwestern United States" or Idaho or Montana or Oregon or Washington or Wyoming or "Pacific States" or Alaska or Hawaii or "Southeastern United States" or Alabama or Arkansas or Florida or Georgia or Louisiana or Mississippi or "North Carolina" or "South Carolina" or "Southwestern United States" or Arizona or California or Colorado or Nevada or "New Mexico" or Texas or Utah or American*).mp.)

HSV-1: Herpes simplex virus type 1.

Methods S1. Description of study methodology, related to STAR Methods.

Methodology	Detailed description
**Data source and search strategy**	- Search was first conducted on September 12, 2021, and updated on April 10, 2023, in PubMed and Embase.<br>- Search strategies included exploded MeSH/Emtree terms and broad terms with no language or time restrictions.<br>- The definition of the United States of America included the District of Columbia and the 50 states:<br>o Alabama, Alaska, Arizona, Arkansas, California, Colorado, Connecticut, Delaware, Florida, Georgia, Hawaii, Idaho, Illinois, Indiana, Iowa, Kansas, Kentucky, Louisiana, Maine, Maryland, Massachusetts, Michigan, Minnesota, Mississippi, Missouri, Montana, Nebraska, Nevada, New Hampshire, New Jersey, New Mexico, New York, North Carolina, North Dakota, Ohio, Oklahoma, Oregon, Pennsylvania, Rhode Island, South Carolina, South Dakota, Tennessee, Texas, Utah, Vermont, Virginia, Washington, West Virginia, Wisconsin, Wyoming. x
**Study selection and inclusion and exclusion criteria**	- Search results were imported into the reference manager Endnote (Thomson Reuters, USA).<br>- Screening was performed in four stages:<br>1. Duplicate publications were identified and excluded.<br>2. Titles and abstracts were screened for relevant and potentially relevant publications.<br>3. Full texts of relevant and potentially relevant publications were retrieved and screened for relevance.<br>4. Bibliographies of relevant publications and reviews were checked for additional potentially relevant publications.<br>- Inclusion criteria were any publication, with a minimum sample size of 10, reporting primary data on any of the following outcome measures:<br>1. HSV-1 seroprevalence as detected by a type-specific diagnostic assay.<br>2. Proportion of HSV-1 detection in clinically diagnosed GUD as detected by standard viral detection and subtyping methods.<br>3. Proportion of HSV-1 detection in laboratory-confirmed genital herpes (as opposed to HSV-2), as detected by standard viral detection and subtyping methods.<br>- Exclusion criteria were:<br>o Case reports, case series, reviews, editorials, commentaries, and qualitative studies.<br>o Measures reporting seroprevalence in infants <6 months-old as their antibodies are maternal in origin.
**Data extraction and data synthesis**	- Extracted variables included: author(s), publication title, year(s) of data collection, publication year, country of origin, city, study site, study design, study sampling procedure, study population and its characteristics (e.g. sex and age), sample size, HSV-1 outcome measures, and diagnostic assay.<br>- For studies including overall sample size, but no individual strata sample sizes, the sample size of each stratum was assumed equal to overall sample size divided by the number of strata in the study.<br>- Stratification hierarchy for seroprevalence, in descending order of preference was population type, then age bracket, and age group:<br>1. Population type classified as:<br>o Healthy general populations: healthy populations such as blood donors, pregnant women, and outpatients with minor health conditions.<br>o Clinical populations: any population with a major clinical condition, or a condition related (potentially) to HSV-1 infection.<br>o Other populations: other populations not satisfying above definitions, or populations with an undetermined risk of acquiring HSV-1, such as HIV-positive patients, sex workers, men who have sex with men, and prisoners.<br>o Specific sub-populations of epidemiological relevance: Sub-populations analyzed separately for HSV-1 seroprevalence due to their epidemiological importance.<br>2. Age category classified as:<br>o Children: <15 years-old individuals.<br>o Adults: ≥15 years-old individuals.<br>3. Age group classified as (groups optimized to best fit reported data):<br>o <10 years old.<br>o 10–19 years old<br>o 20-29 years old.<br>o 30-39 years old.<br>o 40-49 years old.<br>o ≥50 years old.<br>o Mixed age bands.<br>- Stratification hierarchy for GUD and genital herpes in descending order of preference included genital herpes episode status, sex, and study site:<br>1. Genital herpes episode status classified as:<br>o First-episode genital herpes.<br>o Recurrent genital herpes.<br>2. Sex classified as:<br>o Male.<br>o Female.<br>3. Study site stratification classified as:<br>o Hospital.<br>o Sexually transmitted disease clinic.
**Quality assessment**	The Cochrane's approach for risk of bias assessment included:<br><br>- Study's precision classification into low versus high based on the sample size (<100 versus ≥100).<br>- Study's appraisal into low versus high risk of bias was determined using two quality domains:<br>o Sampling method: probability-based versus non-probability based.<br>o Response rate: ≥80% versus <80% or unclear.
**Meta-analyses**	- Meta-analyses were conducted using DerSimonian-Laird random-effects models with inverse variance weighting. The variance of each outcome measure was stabilized using the Freeman-Tukey double arcsine transformation.<br>- Pooled mean HSV-1 seroprevalence was estimated by age, sex, population type, year of data collection, and year of publication.<br>- Pooled mean proportion of HSV-1 detection in genital herpes cases was estimated by sex, genital herpes episode status, year of data collection, and year of publication.<br>- Overall pooled proportion of HSV-1 detection in GUD cases was estimated.<br>- Heterogeneity assessment was based on three complementary metrics:<br>o Cochran's Q statistic to assess existence of heterogeneity in effect size (p-value<0.1 indicated heterogeneity).<br>o I^2 heterogeneity measure to assess the percentage of between-study variation in effect size that is due to true differences in effect size rather than chance.<br>o Prediction interval to describe the distribution of true outcome measures around the pooled mean.
**Meta-regressions**	- Univariable and multivariable random-effects meta-regression analyses using log-transformed proportions were carried out to identify predictors of HSV-1 seroprevalence and HSV-1 proportion in genital herpes.<br>- Factors in the univariable model with a p-value<0.1 were included in the multivariable analysis.<br>- Factors in the multivariable model with a p-value≤0.05 were deemed to be significant predictors.<br>- Variables included in the meta-regression models for HSV-1 seroprevalence were:<br>o Age bracket.<br>o Age group.<br>o Sex.<br>o Population type.<br>o Sample size.<br>o Sampling method.<br>o Response rate.<br>o Year of data collection as a linear term.<br>o Year of data collection as a categorical variable (<1995; 1995-2004; ≥2005).<br>o Year of publication as a linear term.<br>o Year of publication as a categorical variable (<2000; 2000-2009; ≥2010).<br>- Variables included in the meta-regression models for proportion of HSV-1 detection in genital herpes were:<br>o Sex.<br>o Genital herpes episode status.<br>o Year of data collection as a categorical variable (<2000; ≥2000).<br>o Year of data collection as a linear term.<br>- The year of data collection had a few missing values that were imputed by adjusting the year of publication using the median difference with the year of data collection.

ELISA: Enzyme-linked immunosorbent type-specific assay; GUD: Genital ulcer disease; HSV-1: Herpes simplex virus type 1; HSV-2: Herpes simplex virus type 2.

Table S9. Range of quality components applicable to prevalence studies and their applicability to this systematic review study,[42] related to Tables 2 and 4 and STAR Methods.

Risk of bias tool (Hoy, 2012[42])	Relevance to our study
1. Was the study's target population a close representation of the national population in relation to relevant variables?	Met in the study design. The study investigated prevalence in all population groups. The meta-regression analyses also explored the impact of sampling method on observed prevalence. The sampling method was one of the investigated risks of bias domains.
2. Was the sampling frame a true or close representation of the target population?	Met in the study design. Included as the probability-based vs. non-probability-based sampling domain. The meta-regression analyses also explored the impact of sampling method on observed prevalence.
3. Was some form of random selection used to select the sample, OR was a census undertaken?	Met in the study design. Included as the probability-based vs. non-probability-based sampling domain. The meta-regression analyses also explored the impact of sampling method on observed prevalence.
4. Was the likelihood of nonresponse bias minimal?	Met in the study design. Included as the response rate risk of bias domain. The meta-regression analyses also explored the impact of response rate on observed prevalence.
5. Were data collected directly from the subjects (as opposed to a proxy)?	Met in the study design. The inclusion criteria specified that only studies based on biomarkers collected directly from individuals are included.
6. Was an acceptable case definition used in the study?	Met in the study design. A standardized and consistent case definition was used, that of HSV-1 infection.
7. Was the study instrument that measured the parameter of interest shown to have validity and reliability?	Met in the study design. Only studies using valid and reliable diagnostic assays to measure specific biomarkers were included. The assays reported possess acceptable specificity and sensitivity and are commonly used in research and clinical settings. Additionally, the meta-regression analyses explored the impact of assay type on observed prevalence.
8. Was the same mode of data collection used for all subjects?	Met in the study design. It is standard for such infection prevalence studies involving biomarkers, by design, to use a consistent mode of data collection from all subjects recruited for a study, including specimen type and assay type.
9. Was the length of the shortest prevalence period for the parameter of interest appropriate?	Met in the study design. Included studies reported point prevalence measures, that is prevalence based on a cross-sectional survey at a specific and defined time.
10. Were the numerator(s) and denominator(s) for the parameter of interest appropriate?	Met in the study design. The numerator and denominator were defined unambiguously: the number of seropositive HSV-1 cases over the total number of tested subjects for the seroprevalence outcome, and the number of HSV-1 laboratory-confirmed cases over the total number of HSV-1 and HSV-2 laboratory-confirmed cases for the proportion of HSV-1 detection in genital herpes.

HSV-1: Herpes simplex virus type 1; HSV-2: Herpes simples virus type 2.

References

1. Van Der Pol, B., Warren, T., Taylor, S.N., Martens, M., Jerome, K.R., Mena, L., Lebed, J., Ginde, S., Fine, P., and Hook, I.E.W. (2012). Type-specific identification of anogenital herpes simplex virus infections by use of a commercially available nucleic acid amplification test. Journal of Clinical Microbiology *50(11)*, 3466-3471.
2. Binnicker, M.J., Espy, M.J., Duresko, B., Irish, C., and Mandrekar, J. (2017). Automated processing, extraction and detection of herpes simplex virus types 1 and 2: A comparative evaluation of three commercial platforms using clinical specimens. Journal of Clinical Virology *89*, 30-33.
3. Chang, T.W., Fiumara, N.J., and Weinstein, L. (1974). Genital herpes: some clinical and laboratory observations. Journal of the American Medical Association *229(5)*, 544-545.
4. Kalinyak, J.E., Fleagle, G., and Docherty, J.J. (1977). Incidence and distribution of herpes simplex virus types 1 and 2 from genital lesions in college women. J Med Virol *1*, 175-181. 10.1002/jmv.1890010304.
5. Corey, L., Reeves, W.C., Chiang, W.T., Vontver, L.A., Remington, M., Winter, C., and Holmes, K.K. (1978). Ineffectiveness of Topical Ether for the Treatment of Genital Herpes Simplex Virus Infection. New England Journal of Medicine *299*, 237-239. 10.1056/NEJM197808032990507.
6. Vontver, L.A., Reeves, W.C., Rattray, M., Corey, L., Remington, M.A., Tolentino, E., Schweid, A., and Holmes, K.K. (1979). Clinical course and diagnosis of genital herpes simplex virus infection and evaluation of topical surfactant therapy. Am J Obstet Gynecol *133*, 548-554. 10.1016/0002-9378(79)90290-4.
7. Boehm, F.H., Estes, W., Wright, P.F., and Growdon Jr, J.F. (1981). Management of genital herpes simplex virus infection occurring during pregnancy. American Journal of Obstetrics and Gynecology *141(7)*, 735-740.
8. Reichman, R.C., Ginsberg, M., and Barrett-Connor, E. (1982). Controlled trial of oral acyclovir in the therapy of recurrent herpes simplex genitalis. A preliminary report. American Journal of Medicine *73(1 A)*, 338-341.
9. Corey, L., Nahmias, A.J., Guinan, M.E., Benedetti, J.K., Critchlow, C.W., and Holmes, K.K. (1982). A Trial of Topical Acyclovir in Genital Herpes Simplex Virus Infections. New England Journal of Medicine *306*, 1313-1319. 10.1056/NEJM198206033062201.
10. Scher, J., Bottone, E., Desmond, E., and Simons, W. (1982). The incidence and outcome of asymptomatic herpes simplex genitalis in an obstetric population. Am J Obstet Gynecol *144*, 906-909. 10.1016/0002-9378(82)90182-x.
11. Bryson, Y.J., Dillon, M., Lovett, M., Acuna, G., Taylor, S., Cherry, J.D., Johnson, B.L., Wiesmeier, E., Growdon, W., Creagh-Kirk, T., and Keeney, R. (1983). Treatment of first episodes of genital herpes simplex virus infection with oral acyclovir. A randomized double-blind controlled trial in normal subjects. New England Journal of Medicine *308(16)*, 916-921.
12. Bryson, Y., Dillon, M., Bernstein, D.I., Radolf, J., Zakowski, P., and Garratty, E. (1993). Risk of acquisition of genital herpes simplex virus type 2 in sex partners of persons with genital herpes: a prospective couple study. J Infect Dis *167*, 942-946. 10.1093/infdis/167.4.942.
13. Corey, L., Fife, K.H., Benedetti, J.K., Winter, C.A., Fahnlander, A., Connor, J.D., Hintz, M.A., and Holmes, K.K. (1983). Intravenous Acyclovir for the Treatment of Primary Genital Herpes. Annals of Internal Medicine *98*, 914-921. 10.7326/0003-4819-98-6-914.

14. McLaren, C., Corey, L., Dekket, C., and Barry, D.W. (1983). In vitro sensitivity to acyclovir in genital herpes simplex viruses from acyclovir-treated patients. Journal of Infectious Diseases *148(5)*, 868-875.
15. Mertz, G.J., Schmidt, O., Jourden, J.L., Guinan, M.E., Remington, M.L., Fahnlander, A., Winter, C., Holmes, K.K., and Corey, L. (1985). Frequency of acquisition of first-episode genital infection with herpes simplex virus from symptomatic and asymptomatic source contacts. Sex Transm Dis *12*, 33-39. 10.1097/00007435-198501000-00007.
16. Lafferty, W.E., Coombs, R.W., Benedetti, J., Critchlow, C., and Corey, L. (1987). Recurrences after Oral and Genital Herpes Simplex Virus Infection. New England Journal of Medicine *316*, 1444-1449. 10.1056/NEJM198706043162304.
17. Levin, M.J., Judson, F.N., Eron, L., Bryson, Y.J., Corey, L., Murray, M., and Scheer, R.R. (1989). Comparison of intramuscular recombinant alpha interferon (rIFN-2A) with topical acyclovir for the treatment of first-episode herpes genitalis and prevention of recurrences. Antimicrobial Agents and Chemotherapy *33(5)*, 649-652.
18. Ashley, R., Cent, A., Maggs, V., Nahmias, A., and Corey, L. (1991). Inability of enzyme immunoassays to discriminate between infections with herpes simplex virus types 1 and 2. Annals of internal medicine *115*, 520-526.
19. Koelle, D.M., Benedetti, J., Langenberg, A., and Corey, L. (1992). Asymptomatic reactivation of herpes simplex virus in women after the first episode of genital herpes. Ann Intern Med *116*, 433-437. 10.7326/0003-4819-116-6-433.
20. Lebwohl, M., Sacks, S., Conant, M., Connor, J., Douglas Jr, J.M., Eron, L., Marlowe, S., Mendelson, J., Chen, V., Bradstreet, P., et al. (1992). Recombinant alpha-2 interferon gel treatment of recurrent herpes genitalis. Antiviral Research *17(3)*, 235-243.
21. Mertz, G.J., Benedetti, J., Ashley, R., Selke, S.A., and Corey, L. (1992). Risk factors for the sexual transmission of genital herpes. Ann Intern Med *116*, 197-202. 10.7326/0003-4819-116-3-197.
22. Benedetti, J., Corey, L., and Ashley, R. (1994). Recurrence rates in genital herpes after symptomatic first-episode infection. Annals of Internal Medicine *121(11)*, 847-854.
23. Skinner, G.R.B., Turyk, M.E., Benson, C.A., Wilbanks, G.D., Heseltine, P., Galpin, J., Kaufman, R., Goldberg, L., Hartley, C.E., and Buchan, A. (1997). The efficacy and safety of Skinner herpes simplex vaccine towards modulation of herpes genitalis; Report of a prospective double blind placebo controlled trial. Medical Microbiology and Immunology *186(1)*, 31-36.
24. Benedetti, J.K., Zeh, J., and Corey, L. (1999). Clinical reactivation of genital herpes simplex virus infection decreases in frequency over time. Ann Intern Med *131*, 14-20. 10.7326/0003-4819-131-1-199907060-00004.
25. Lafferty, W.E., Downey, L., Celum, C., and Wald, A. (2000). Herpes simplex virus type 1 as a cause of genital herpes: impact on surveillance and prevention. J Infect Dis *181*, 1454-1457. 10.1086/315395.
26. Reyes, M., Shaik, N.S., Graber, J.M., Nisenbaum, R., Wetherall, N.T., Fukuda, K., and Reeves, W.C. (2003). Acyclovir-resistant genital herpes among persons attending sexually transmitted disease and human immunodeficiency virus clinics. Archives of Internal Medicine *163(1)*, 76-80.
27. Brown, Z.A., Wald, A., Morrow, R.A., Selke, S., Zeh, J., and Corey, L. (2003). Effect of serologic status and cesarean delivery on transmission rates of herpes simplex virus from mother to infant. Jama *289(2)*, 203-209.

28. Solomon, L., Cannon, M.J., Reyes, M., Graber, J.M., Wetherall, N.T., and Reeves, W.C. (2003). Epidemiology of recurrent genital herpes simplex virus types 1 and 2. Sexually Transmitted Infections *79(6)*, 456-459.
29. Roberts, C.M., Pfister, J.R., and Spear, S.J. (2003). Increasing proportion of herpes simplex virus type 1 as a cause of genital herpes infection in college students. Sexually Transmitted Diseases *30(10)*, 797-800.
30. Sizemore Jr, J.M., Lakeman, F., Whitley, R., Hughes, A., and Hook, I.E.W. (2006). The spectrum of genital herpes simplex virus infection in men attending a sexually transmitted disease clinic. Journal of Infectious Diseases *193(7)*, 905-911.
31. Handsfield, H.H., Warren, T., Werner, M., and Phillips, J.A. (2007). Suppressive therapy with valacyclovir in early genital herpes: A pilot study of clinical efficacy and herpes-related quality of life. Sexually Transmitted Diseases *34(6)*, 339-343.
32. Fife, K.H., Meng, T.C., Ferris, D.G., and Liu, P. (2008). Effect of resiquimod 0.01% gel on lesion healing and viral shedding when applied to genital herpes lesions. Antimicrobial Agents and Chemotherapy *52(2)*, 477-482.
33. Wald, A., Richards, J., Krantz, E., and Selke, S. (2008). Healthcare seeking and sexual behavior among patients with symptomatic newly acquired genital herpes. Sexually Transmitted Diseases *35(12)*, 1015-1021.
34. Horowitz, R., Aierstuck, S., Williams, E.A., and Melby, B. (2010). Herpes simplex virus infection in a university health population: Clinical manifestations, epidemiology, and implications. Journal of American College Health *59(2)*, 69-74.
35. Van Wagoner, N.J., Geisler, W.M., Sizemore Jr, J.M., Whitley, R., and Hook, I.E.W. (2010). Herpes simplex virus in African American heterosexual males: The roles of age and male circumcision. Sexually Transmitted Diseases *37(4)*, 217-222.
36. Katsumata, K., Weinberg, A., Chono, K., Takakura, S., Kontani, T., and Suzuki, H. (2012). Susceptibility of herpes simplex virus isolated from genital herpes lesions to ASP2151, a novel helicase-primase inhibitor. Antimicrobial Agents and Chemotherapy *56(7)*, 3587-3591.
37. Tyring, S., Wald, A., Zadeikis, N., Dhadda, S., Takenouchi, K., and Rorig, R. (2012). ASP2151 for the treatment of genital herpes: A randomized, double-blind, placebo- and valacyclovir-controlled, dose-finding study. Journal of Infectious Diseases *205(7)*, 1100-1110.
38. Young, S., Van Der Pol, B., Taylor, S., Fife, K., Hook, E., Patel, R., Ding, K., Hemyari, P., Duncan, J., Tang, S., et al. (2015). Evaluation of the cobas HSV 1 and 2 test for the detection of HSV from clinician-collected anogenital lesion swab specimens compared with elvis HSV id and d3 typing test and sanger sequencing. Sexually Transmitted Infections *2)*, A129.
39. Faron, M.L., Ledeboer, N.A., Patel, A., Beqa, S.H., Yen-Lieberman, B., Kohn, D., Leber, A.L., Mayne, D., Northern, W.I., and Buchan, B.W. (2016). Multicenter evaluation of meridian bioscience hsv 1&2 molecular assay for detection of herpes simplex virus 1 and 2 from clinical cutaneous and mucocutaneous specimens. Journal of Clinical Microbiology *54(8)*, 2008-2013.
40. Dabestani, N., Katz, D.A., Dombrowski, J., Magaret, A., Wald, A., and Johnston, C. (2019). Time Trends in First-Episode Genital Herpes Simplex Virus Infections in an Urban Sexually Transmitted Disease Clinic. Sexually Transmitted Diseases *46(12)*, 795-800.

41. Page, M.J., McKenzie, J.E., Bossuyt, P.M., Boutron, I., Hoffmann, T.C., Mulrow, C.D., Shamseer, L., Tetzlaff, J.M., Akl, E.A., and Brennan, S.E. (2021). The PRISMA 2020 statement: an updated guideline for reporting systematic reviews. International journal of surgery *88*, 105906.
42. Hoy, D., Brooks, P., Woolf, A., Blyth, F., March, L., Bain, C., Baker, P., Smith, E., and Buchbinder, R. (2012). Assessing risk of bias in prevalence studies: modification of an existing tool and evidence of interrater agreement. Journal of clinical epidemiology *65*, 934-939.
